# Down‐regulation of Lon protease 1 lysine crotonylation aggravates mitochondrial dysfunction in polycystic ovary syndrome

**DOI:** 10.1002/mco2.396

**Published:** 2023-10-09

**Authors:** Yuan Xie, Shuwen Chen, Zaixin Guo, Ying Tian, Xinyu Hong, Penghui Feng, Qiu Xie, Qi Yu

**Affiliations:** ^1^ Department of Obstetrics and Gynecology National Clinical Research Center for Obstetric & Gynecologic Diseases State Key Laboratory for Complex Severe and Rare Diseases Peking Union Medical College Hospital Chinese Academy of Medical Sciences & Peking Union Medical College Peking Union Medical College Hospital (Dongdan Campus) Beijing China; ^2^ Department of Medical Research Center State Key Laboratory for Complex Severe and Rare Diseases Peking Union Medical College Hospital Chinese Academy of Medical Science and Peking Union Medical College Beijing China

**Keywords:** LONP1, lysine crotonylation, mitochondrial dysfunction, polycystic ovary syndrome

## Abstract

Polycystic ovary syndrome (PCOS) is a prevalent reproductive endocrine disorder, with metabolic abnormalities and ovulation disorders. The post‐translational modifications (PTMs) are functionally relevant and strengthen the link between metabolism and cellular functions. Lysine crotonylation is a newly identified PTM, the function of which in PCOS has not yet been reported. To explore the molecular mechanisms of crotonylation involved in the abnormalities of metabolic homeostasis and oocyte maturation in PCOS, by using liquid chromatography‐tandem mass spectrometry analysis, we constructed a comprehensive map of crotonylation modifications in ovarian tissue of PCOS‐like mouse model established by dehydroepiandrosterone induction. The crotonylation levels of proteins involved in metabolic processes were significantly decreased in PCOS ovaries compared to control samples. Further investigation showed that decrotonylation of Lon protease 1 (LONP1) at lysine 390 was associated with mitochondrial dysfunction in PCOS. Moreover, LONP1 crotonylation levels in PCOS were correlated with ovarian tissue oxidative stress levels, androgen levels, and oocyte development. Consistently, down‐regulation of LONP1 and LONP1 crotonylation levels were also observed in the blood samples of PCOS patients. Collectively, our study revealed a mechanism by which the decrotonylation of LONP1 may attenuate its activity and alter follicular microenvironment to affect oocyte maturation in PCOS.

## Graphical abstract

This study aimed to construct a comprehensive map of crotonylation modifications in the ovarian tissue of PCOS‐like mice. Additionally, the association between LONP1‐K390 decrotonylation and mitochondrial dysfunction in PCOS was investigated. Overall, our study revealed a mechanism by which the decrotonylation of LONP1 may attenuate its activity and mitochondrial function and alter follicular micro‐environment to affect oocyte development in PCOS

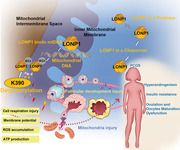

## 1 INTRODUCTION

Polycystic ovary syndrome (PCOS) is one of the most prevalent and intricate endocrine disorders observed in women within the reproductive age group.^1^ The condition is distinguished by persistent anovulation, hyperandrogenism, and escalating metabolic irregularities, including insulin resistance (IR), hyperinsulinemia, and hyperlipidemia, which intensify with advancing age.^2^ Metabolic disorders contribute to PCOS‐related infertility by disrupting ovarian micro‐environment homeostasis, which in turn interferes with follicle development and oocyte maturation.^3^, ^4^ However, the heterogeneous nature of PCOS, with multiple potential etiologies, indicates that disease pathogenesis may be influenced by both genetic and environmental factors. The post‐translational modifications (PTMs) are functionally relevant and strengthen the link between metabolism and cellular functions. A recent research reported that the changes in lysine acetylation in a key enzyme acetyl‐CoA acetyltransferase 1 in PCOS granulosa cells were correlated with oocyte quality and embryo development efficiency in the clinic.^5^ PCOS is therefore linked to epigenetics, particularly protein PTMs.^6^

A newly identified PTM in histone and non‐histone proteins, lysine crotonylation (Kcr), mainly occurs on the ε‐amino group.^7^, ^8^, ^9^ Kcr modification is associated with biological processes (glycolysis, neural development, differentiation of human embryonic stem cells, and sperm development)^10^, ^11^, ^12^ and several diseases (kidney disease,^13^ cardiovascular disease, tumors).^14^ A growing number of research have indicated that modifications in crotonylation of core proteins may lead to alterations to their activities and functions, potentially exacerbating or ameliorating the disease phenotype. According to recent publications, the crotonylation of glyceraldehyde‐3‐phosphate dehydrogenase resulted in a decline in its enzymatic activity, ultimately causing a decrease in glycolysis during the process of endodermal differentiation from human embryonic stem cell.^11^ The loss of acyl‐CoA oxidase 1 and acyl‐CoA oxidase 3, the key enzymes producing tart aryl‐CoA, causes decreased Kcr, impairing the differentiation of pluripotent embryonic stem cells into mesoderm and endoderm; hence, it triggers a metabolic transition from glycolysis to oxidative phosphorylation.^15^ Additionally, regulation of the specific cytoskeleton and the Kcr sites of the mitochondrial protein isocitrate dehydrogenase 3A has been found to protect mice against ischemia–reperfusion‐induced cardiac dysfunction.^16^

Several studies have demonstrated that the key differential genes in PCOS are mainly encoded in pathways related to regulatory metabolism, such as inflammation^17^ and immune response, insulin signaling, carbohydrate and lipid metabolism, hormonal regulation, and mitochondrial activity.^5^, ^18^ In addition to producing adenosine 5'‐triphosphate (ATP), through oxidative phosphorylation, mitochondria release reactive oxygen species (ROS). The production of ROS is important for cellular functions such as proliferation, metabolism, gene expression, and immune response.^19^, ^20^ In recent studies, it has been shown that mitochondrial dysfunction induces PCOS‐related metabolic comorbidities (obesity, IR, and hyperandrogenism) through increased ROS production and oxidative stress.^20^

In this study, we are committed to ascertain the regulatory function of epigenetic factors in the pathogenesis of PCOS and associated infertility and to explore the impact of crotonylation modification on mitochondrial function and oocyte maturation in PCOS. Our study suggests, for the first time, that function and activity changes in specific mitochondrial protein Lon protease 1 (LONP1) may be linked to oxidative stress in oocytes of PCOS mice. In addition, it was demonstrated that mutation in core Kcr site of LONP1 may exacerbate mitochondrial dysfunction.

## 2 RESULTS

### 2.1 Analysis of PCOS phenotype in dehydroepiandrosterone‐induced PCOS mouse model

**FIGURE 1:**
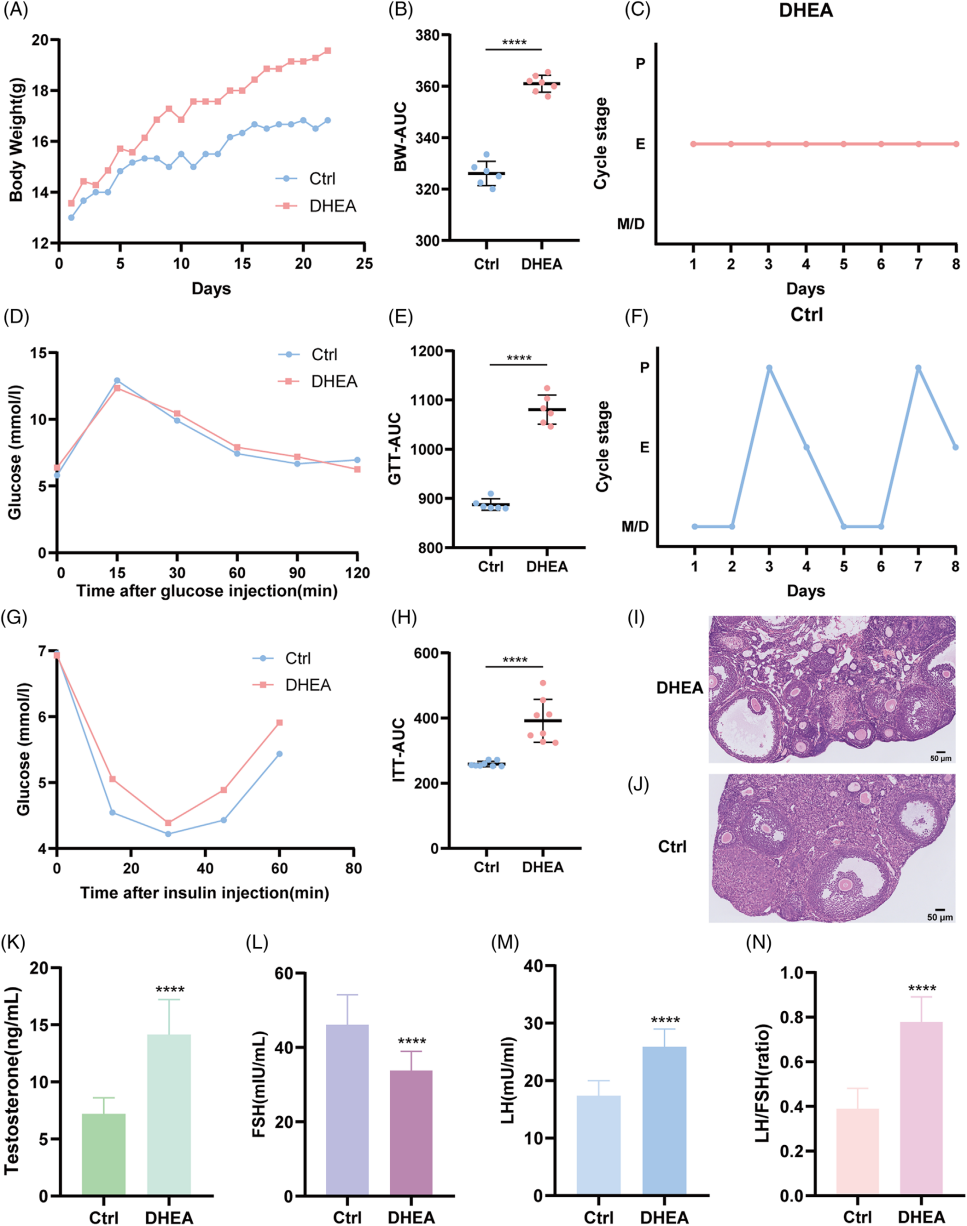
Phenotypic evaluation of normal and dehydroepiandrosterone (DHEA)‐induced mice. (A and B) Comparison of body weight curves and area under the curve (AUC) of body weight changes before and after DHEA administration in mice. (C and F) Representative estrous cyclicity assessment in 5‐week‐old female mice for eight consecutive days by vaginal cytology. The graph shows representative cycles from two groups. Each dot represents a day. M, metestrus; D, diestrus; P, proestrus; E, estrus. (D and G) Glucose tolerance tests and insulin resistance experiments in mice. (E and H) The difference between the two groups was determined by calculating AUC. (I and J) Ovarian morphology by hematoxylin and eosin (H&E )staining in control and DHEA‐induced mice. (K–N) Analysis of serum concentrations of testosterone (K), follicle‐stimulating hormone (FSH) (L), luteinizing hormone (LH) (M), and LH/FSH (N) as measured by enzyme‐linked immunoassay (ELISA) (*n* = 6/group). All error bars, mean values ± SD, and *p*‐values were determined by unpaired two‐tailed Student's *t*‐test of *n* = 3 independent biological experiments. ^*^ *p* < 0.05; ^**^ *p* < 0.01; ^***^ *p* < 0.001; ^****^ *p* < 0.0001.

To determine the role of lysine crotonylation in PCOS development, dehydroepiandrosterone (DHEA)‐induced PCOS mice were modeled according to a previous study.^21^ Body weight (BW), estrous cycle, sex hormone levels, and ovarian tissue morphology were monitored. Both control and DHEA‐modeled mice were well developed before the experimental intervention, and there were no significant differences in their behavior and physical signs. No significant difference was observed in initial BW between the two groups (13 ± 1.27 g and 13.57 ± 1.40 g, respectively; *p* > 0.9999). After 21 days of DHEA administration, the BW data reflected differences (16.83 ± 1.17 g and 19.57 ± 1.13 g; *p* < 0.0001). This also reflects a significant difference from the area under the curve (AUC) of weight gain. The control group had an AUC of 326.08 ± 4.75, and the model group's AUC was 361 ± 3.30 (*p* < 0.0001) (Figure 1A,B). Control mice showed normal estrous cyclicity, whereas mice treated with DHEA displayed abnormal cycles. Figure 1C,F shows representative cyclicities of mice in the two groups. Glucose tolerance test (GTT) experiments showed no significant difference in fasting glucose (5.81 ± 1.02 vs. 6.39 ± 0.85), but the AUC of the GTT in the model group was significantly higher than that of the control group (887.67 ± 11.46 vs. 1080.5 ± 29.51; *p* < 0.0001) (Figure 1D,E). Similar results were observed with insulin tolerance test AUC (258.96 ± 8.31 vs. 391.61 ± 66.05; *p* < 0.0001) (Figure 1G,H). The hematoxylin and eosin (H&E)‐stained ovarian tissue showed numerous corpora lutea and growing follicles. Conversely, there was little or no luteum in H&E‐stained PCOS mouse ovaries. In addition, a large number of vesicular follicles, a significant reduction in the granulosa cell layer, and prominent polycystic‐like changes were seen in these ovaries (Figure 1I,J). Mouse serum sex hormones were determined by chemiluminescence, and the results showed that serum testosterone (T), follicle‐stimulating hormone (FSH), luteinizing hormone (LH), and LH/FSH concentrations in the PCOS mouse group were significantly higher (Figure 1K–N). Thus, we showed that the DHEA‐induced PCOS‐like model mimics the phenotypes of PCOS obesity, abnormal ovulation, abnormal glucose, and insulin tolerance.

### 2.2 Bioinformatics analysis of differentially crotonylated proteins and sites in PCOS ovarian tissues

**FIGURE 2:**
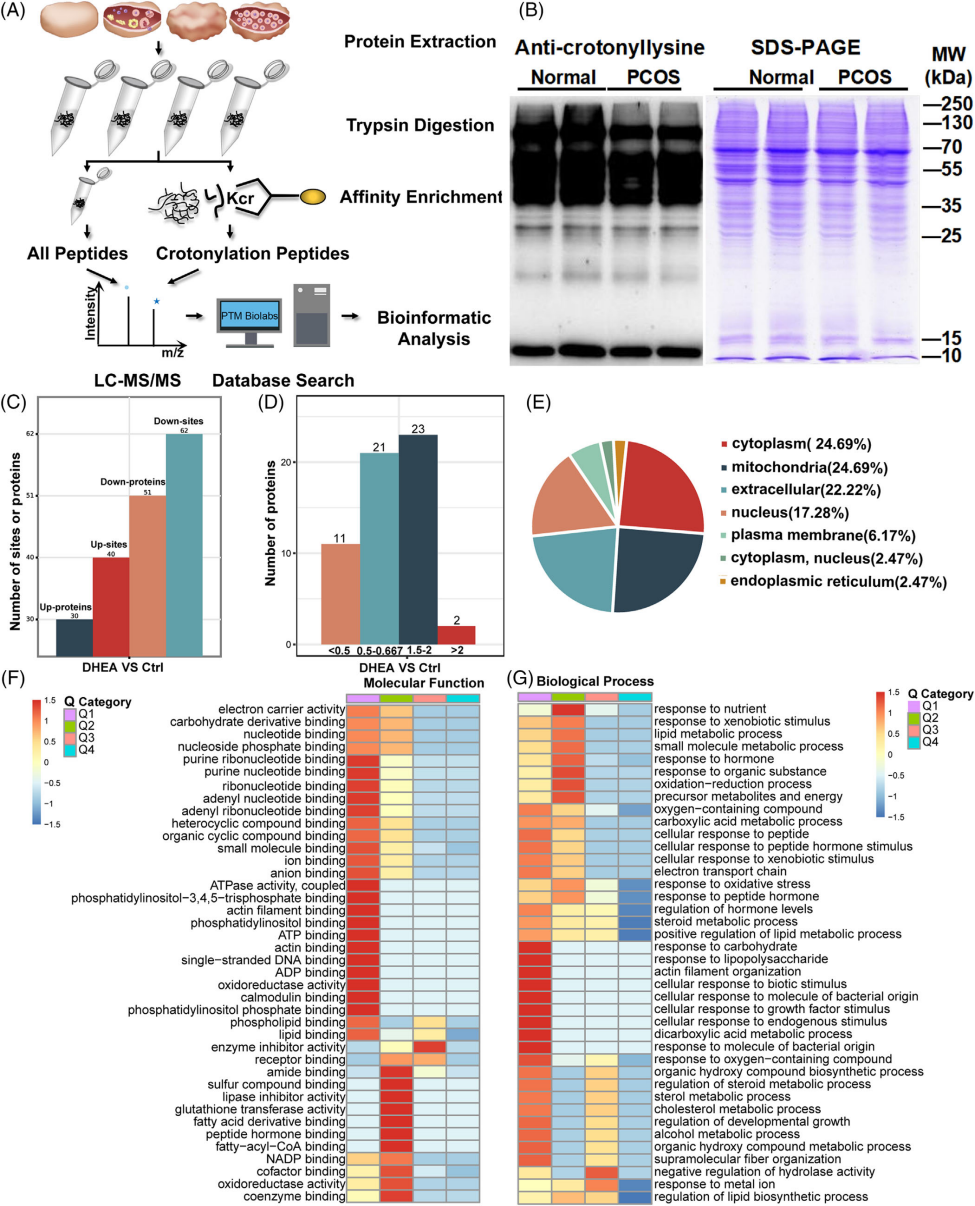
Identification and bioinformatics analysis of differentially crotonylated proteins and sites. (A) Detection of lysine crotonylation in ovarian tissues of polycystic ovary syndrome (PCOS) and normal mice. Diagram of ovary tissue collection, protein lysis, and liquid chromatography‐tandem mass spectrometry (LC‐MS/MS) of all protein and crotonylation peptides. The elements used (A) were finished with Adobe Illustrator and Adobe Photoshop and authorized by Hefei Sondii Media Co., Ltd. (B) Western blot analysis of ovary tissues from two groups by anti‐crotonyl lysine antibody. Each sample was combined and examined. The loading was controlled by Coomassie blue staining. (C) Number of differentially crotonylated sites and proteins in the PCOS group when compared with the control group. The differential expression multiples were more than 1.5. (D) Quantitative classification and numbers of differentially crotonylated proteins in each quantified part. The differentially modified proteins were divided into four quantified parts according to their differential expression multiples: Q1 (0 < ratio ≤ 1/2), Q2 (1/2 < ratio ≤ 1/1.5), Q3 (1.5 < ratio ≤ 2), and Q4 (ratio > 2). (E) The predication of subcellular localization of differentially crotonylated proteins using WoLF PSORT software. (F and G) Clustering analysis including molecular function analysis (F) and biological process analysis (G) based on Gene Ontology (GO) enrichment database of quantitative differentially crotonylated proteins in each Q group.

In order to construct a relatively complete map of crotonylation modifications in PCOS ovarian tissue, we pooled 20 PCOS and normal mouse ovaries separately for quantitative proteomic analysis with liquid chromatography‐tandem mass spectrometry assay. Initially, we screened the most obvious forms of protein PTMs in PCOS mice compared with normal mice, including acetylation, succinylation, acrylation, malonylation, and crotonylation. It was observed that the ovarian tissue of mice in the PCOS group had a significant overall down‐regulation of crotonylation modification (Figure 2B). Hence, in order to comprehensively ascertain the alterations in protein Kcr within PCOS ovarian tissues, a quantitative investigation and analysis of Kcr modification omics were conducted following the outlined procedure depicted in Figure 2A. The MS data validation is shown in Figure S1. The majority of peptides exhibited a length distribution spanning from seven to 13, aligning with the typical characteristics of tryptic peptides (Figure S1A). This observation suggests that the sample preparation procedure adhered to standard protocols. Meanwhile, relative standard deviation values of more than 70% of the samples (from both groups) were less than or equal to 20%, which indicated that the method had good stability and repeatability, and the data obtained were reliable (Figure S1B).

In order to establish a robust level of certainty in the outcomes, we employed a filtration process on the identification data, utilizing a localization probability threshold of >0.75. Consequently, a comprehensive tally of 16,800 crotonylation sites distributed across 3934 proteins was successfully identified. Notably, among these sites, a subset of 11,020 sites spanning 3031 proteins provided quantitative information, as depicted in Figure S1C. Among the proteins that were differentially crotonylated, a total of 102 sites were identified on 81 distinct proteins, exhibiting expression multiples greater than 1.5 between the PCOS and normal groups. Specifically, 40 sites on 30 proteins displayed elevated levels of crotonylation, while 62 sites on 51 proteins exhibited decreased levels of crotonylation within the PCOS group (Figure 2C). To determine the functional correlation of proteins with different differential expression multiples, we divided the differentially expressed proteins into four parts, Q1 to Q4 (Q1 [0 < ratio ≤ 1/2], Q2 [1/2 < ratio ≤ 1/1.5], Q3 [1.5 < ratio ≤ 2], and Q4 [ratio > 2]), according to their differential expression multiples (Figure 2D). We conducted a comprehensive bioinformatics analysis on proteins that possess quantitatively informative loci, encompassing protein annotation, functional classification, functional enrichment, and clustering analysis based on functional enrichment.

Figure 2E illustrates how Kcr regulates the cellular functions by determining its subcellular localization. The results showed that these distinguished crotonylated proteins were distributed extensively, mainly in the mitochondria (24.69%), cytoplasm (24.69%), extracellular space (22.22%), and nucleus (17.29%) (Figure 2E). To gain further insights into the cellular pathways underlying Kcr in PCOS, we conducted a comparative analysis of differentially expressed proteins across the two groups. Subsequently, we performed enrichment analysis at three distinct levels, namely, Gene Ontology (GO) classification, Kyoto Encyclopedia of Genes and Genomes (KEGG) pathway, and protein domain. Fisher's exact test was employed for the enrichment test, and the resulting *p*‐value, as depicted in the bubble chart, indicated the functional classification and pathway enrichment of differentially expressed proteins (*p* < 0.05). An analysis of the 20 most significantly enriched categories is presented in a bubble plot.^6^ Based on the GO functional taxonomies, the proteins in the PCOS group with notable changes in crotonylation levels were primarily involved in metabolic processes, including protein synthesis, carbohydrate metabolism, glucose utilization, lipid metabolism, and regulation of protease and ATPase (Figure S1D,E). A considerable part of their cellular components were related to mitochondria and intracellular vesicles (Figure S1F). Further functional enrichment analysis was performed using the KEGG database, and several metabolically related pathways showed a significant correlation with differentially expressed proteins. Specifically, butanoate, tryptophan, glutathione, pyruvate, cholesterol, and carbon metabolism are involved. The citrate cycle (tricarboxylic acid [TCA] cycle), peroxisome proliferators‐activated receptors signaling pathway and fatty acid degradation (Figure S1G).

Furthermore, GO classification and cluster analysis were conducted for each Q group. According to the analysis results at the molecular function level, Q1 protein was mainly concentrated in binding with ATPase activity, coupled ATP, and adenosine diphosphate binding. Q2 was mainly related to glutathione transferase activity and nicotinamide adenine dinucleotide phosphate binding (Figure 2F). Most of the biological processes, including lipid metabolic processes, responses to oxidative stress, and carbohydrate processes, were related to Q1 and Q2, especially the proteins of Q1 (Figure 2G).

### 2.3 Identification of core protein LONP1 and its critical crotonylation site

**FIGURE 3:**
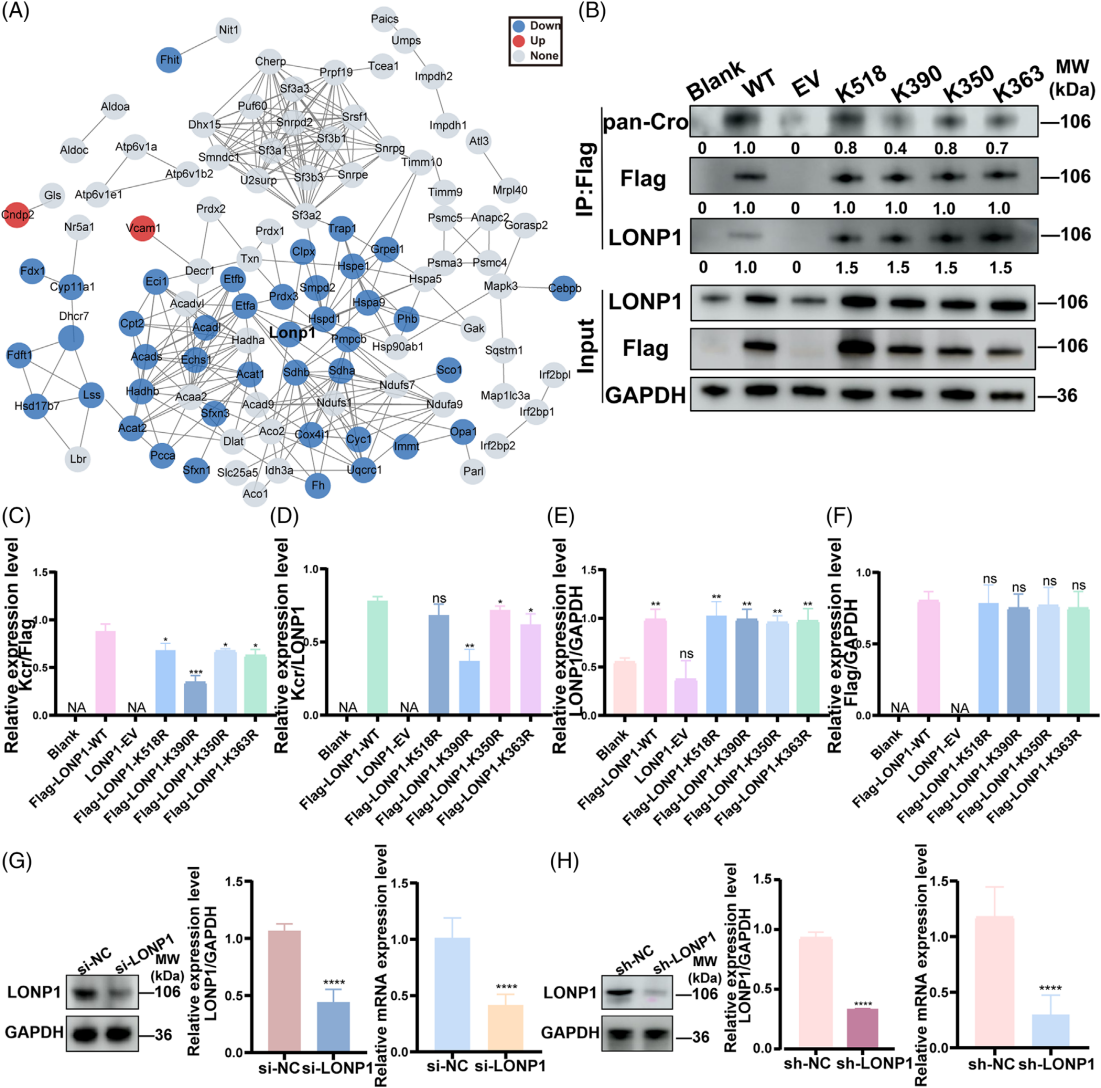
Down‐regulation of Lon protease 1 (LONP1) lysine crotonylation at K390 (A) protein‐protein interaction (PPI) network on the basis of the STRING database (version 10.5) was visualized in Cytoscape. Representative mitochondrial proteins enriched by lysine crotonylation are illustrated. (B) The crotonylation level of LONP1 in untreated normal cells (blank), empty vector (EV), Flag‐tagged wild‐type LONP1 (WT), and K518R, K390R, K350R, and K363R mutant Chinese hamster ovary (CHO) cells. The amount of pan‐Cro, Flag, and LONP1 are shown at below. (C and D) The ratio of pan‐Cro to Flag (C), and the ratio of pan‐Cro to LONP1 (D) in different conditions in immunoprecipitation (IP) assays are shown. The statistical significance was compared separately to the WT group. NA, not available. (E and F) The amounts of LONP1/GAPDH and Flag/GAPDH were calculated as shown in (E) and (F). The statistical significance was compared separately to the blank group. (G and H) Transient and stable knockdown of endogenous LONP1 in CHO cells was performed and verified via western blotting (WB) and reverse transcriptase polymerase chain reaction (RT‐PCR) analysis. All error bars, mean values ± SD, and *p*‐values were determined by unpaired two‐tailed Student's *t*‐test of *n* = 3 independent biological experiments. ^*^ *p* < 0.05; ^**^ *p* < 0.01; ^***^ *p* < 0.001; ^****^ *p* < 0.0001.

The results of the above bioinformatics analysis showed that the discrepant crotonylated proteins were mainly concentrated in metabolism‐related pathways, which are closely linked to mitochondrial function and oxidative stress. To figure out target molecules that undergo differential crotonylation modifications and are closely related to mitochondrial function, we selected several differentially expressed proteins associated with mitochondria, including LONP1, acetyl‐CoA acetyltransferase 1, heat shock protein family D member 1, peroxiredoxin 3, optic atrophy 1, hydroxyacyl‐CoA dehydrogenase trifunctional multienzyme complex subunit alpha, and succinate dehydrogenase, for protein–protein interaction (PPI) analysis to explore the interaction relationship between each protein PPI, which showed that crotonylated mitochondrial proteins possess a degree of crosstalk, and 128 proteins were identified as nodes connected by 107 proteins. Forty percent were down‐regulated and only two proteins were up‐regulated (Figure 3A). In addition, we found four proteins with 50 crotonylation sites, myosin 1b, ribosomal protein S11, LONP1, and cytochrome P450 family 11 subfamily A member 1, which showed more than 2.5‐fold down‐regulation of crotonylation levels, suggesting a potential function in the development of PCOS. Among them, LONP1, mitochondrial matrix protein LONP1, is associated with ATP enzymes with various cellular activities and is most closely involved in oxidative stress regulation. Based on the results so far, we proposed that LONP1 may play an essential role in the pathogenesis of PCOS and become a potential diagnostic or therapeutic target.

Based on quantitative analysis, four plasmids with LONP1 Kcr site mutations were generated and labeled with a 3xflag tag. Specifically, LONP1‐K518R, K390R, K350R, and K363R were used to simulate the decrotonylation of LONP1. These plasmids were then transfected into Chinese hamster ovary (CHO) cells to identify the core crotonylation site in LONP1, after which we performed co‐immunoprecipitation (Co‐IP) assay between LONP1 and Kcr. The complexes containing the LONP1 protein were immunoprecipitated with the flag‐coupled beads, and the expression of crotonylation was observed by western blotting (WB). The results revealed that LONP1‐K390R exhibited the most significant down‐regulation of crotonylation when compared to other mutant sites (Figures 3B–F and S2A). To further verify the potential impact of decrotonylation at K390 to LONP1 activity and functions, we determined the in vitro enzymatic activities of these mutants expressed in CHO cells. In order to exclude or minimize the impact of endogenous LONP1, as comprehensive genetic knockout of the LONP1 protease was not viable in cultured cells and led to embryonic lethality in mice.^22^ To address this issue, we employed LOPN1 small interfering RNAs (siRNAs) and short hairpin RNAs (shRNAs) (Figure S2B) to knockdown (KD) endogenous LOPN1. Both protein and mRNA levels of LOPN1 were significantly down‐regulated in LOPN1 KD CHO cells (Figure 3G,H).

### 2.4 LONP1 decrotonylation of K390 failed to rescue mitochondrial impair in CHO KD cells

**FIGURE 4:**
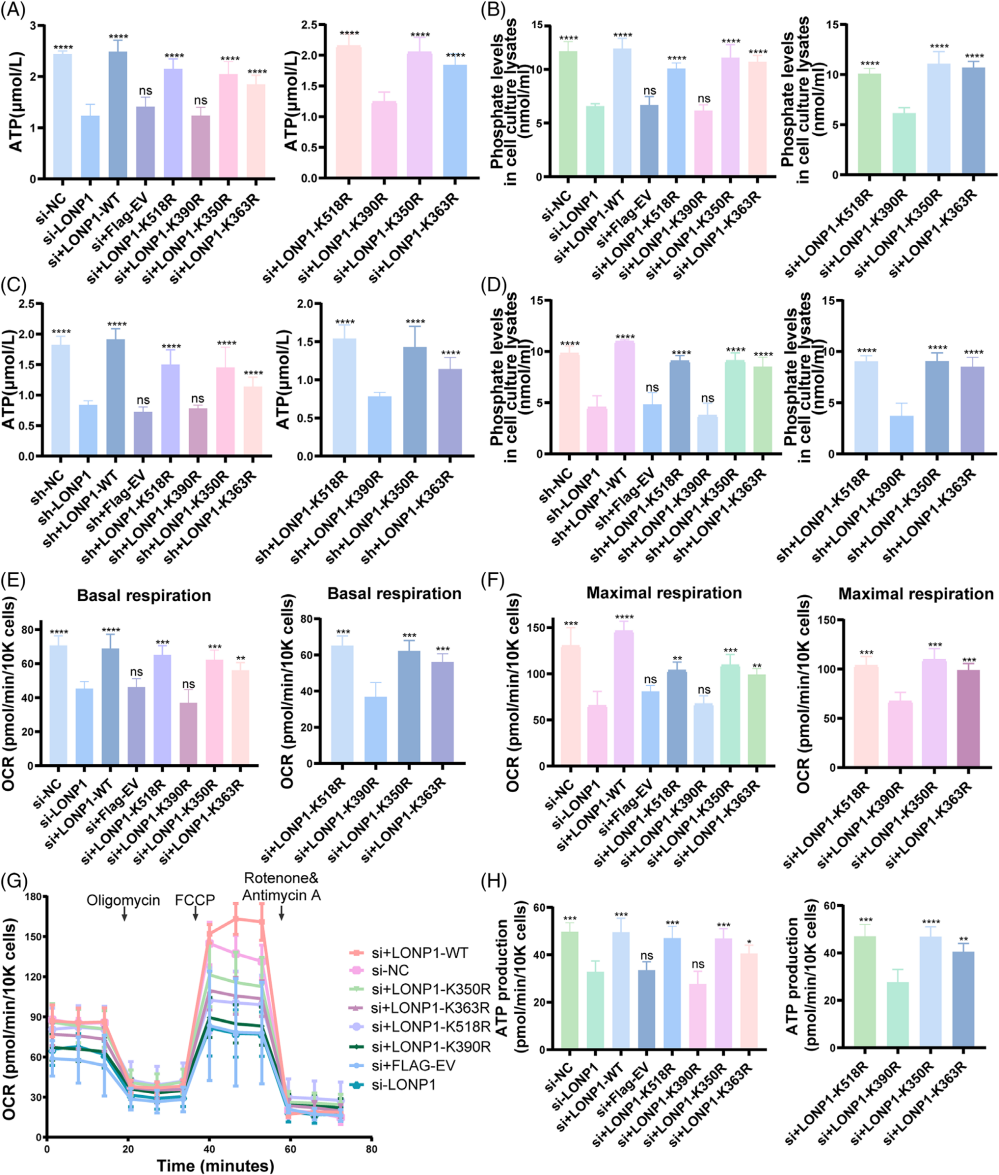
Lon protease 1 (LONP1) decrotonylation of K390 failed to rescue cell respiration and activity impairment in Chinese hamster ovary (CHO) knockdown cells. (A and C) Detection of intracellular ATP levels in transient and stable knockdown CHO cells. (B and D) The rates of ATP hydrolysis of the LONP1 forms were assessed using the malachite green phosphate assay in each group in transient and stable knockdown CHO cells. (E–H) Measurement of mitochondrial oxygen consumption ratio (OCR) and ATP production level in over‐expressed plasmids containing empty vector, WT LONP1, and LONP1‐K518R, K390R, and K350R mutants in si‐LONP1 CHO cells by XFe96 flux analyzer. The statistical significance was compared separately to the si‐LONP1 group and K390R group. All error bars, mean values ± SD, and *p*‐values were determined by unpaired two‐tailed Student's *t*‐test of *n* = 3 independent biological experiments. ^*^ *p* < 0.05; ^**^ *p* < 0.01; ^***^ *p* < 0.001; ^****^ *p* < 0.0001.

To avoid the KD effects of siRNAs and shRNA targeting LONP1, the target sites of LONP1 siRNAs and shRNA in wild‐type (WT) and mutated LONP1 plasmids were mutated synonymously. We transfected the LONP1 KD cells with different types of LONP1 mutations, including LONP1‐K518R, K390R, K350R, and K363R, and then examined the levels of phosphate and ATP. The results show that both phosphate and ATP levels are not recovered in LONP1 KD cells with the over‐expression of LONP1‐K390R rather than LONP1‐K518R, K350R, and K363R (Figure 4A–D). Furthermore, we performed the Seahorse testing to evaluate the ability of cell mitochondria stress. Our findings indicate a remarkable reduction in the oxygen consumption rate (OCR) during the cell mitochondria stress test under different conditions (basal respiration and maximal respiration) in LONP1 KD CHO cells (Figure 4E,F). Compared to the WT LONP1, LONP1 with K390R mutation could not rescue OCR during the cell mitochondrial stress test and ATP production (Figure 4G,H). In contrast, the other types of LONP1 mutations, including K350R, K363R, and K518R, can partially recover the deficiency of mitochondria evaluated by seahorse assay in LONP1 KD cells.

**FIGURE 5:**
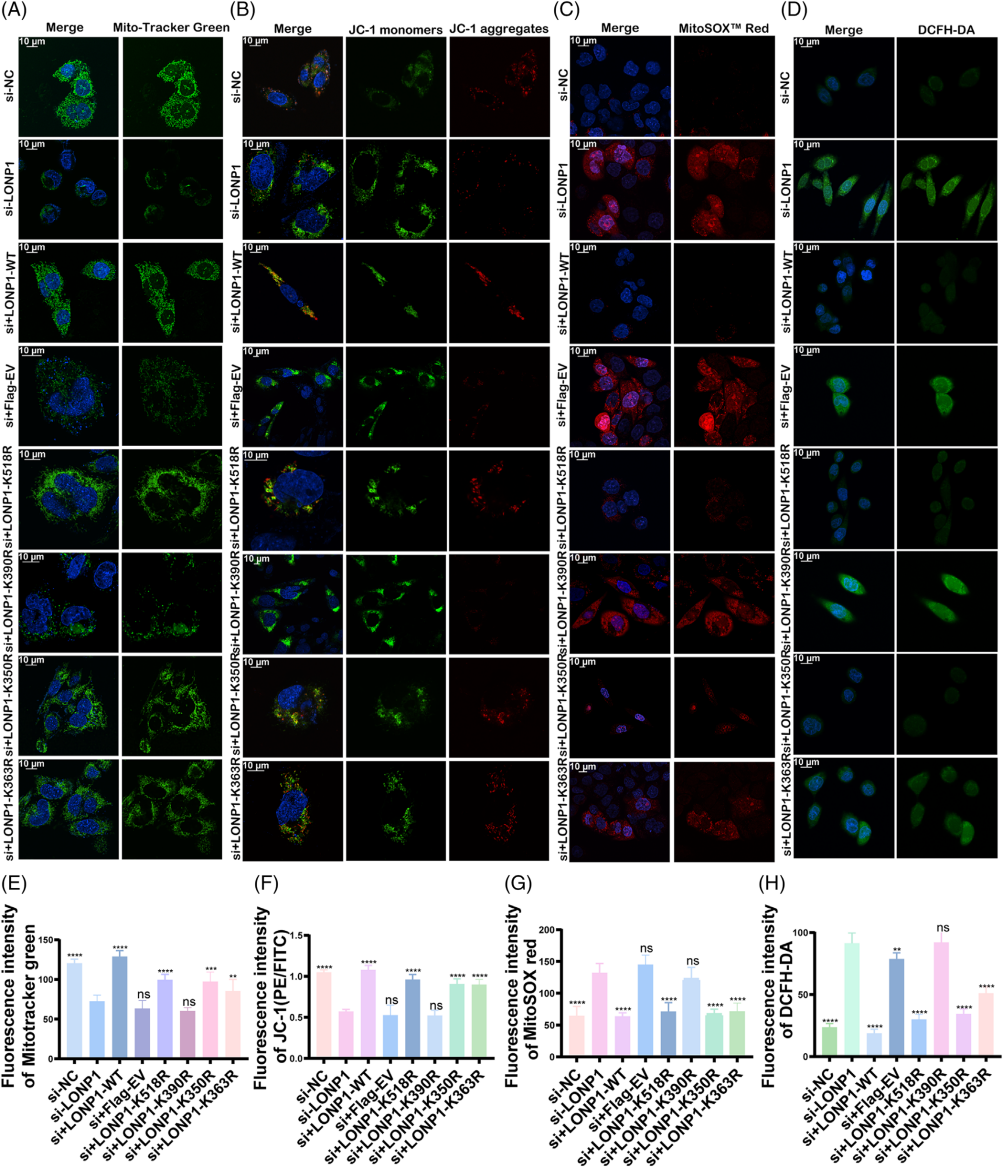
Down‐regulation of Lon protease 1 (LONP1) lysine crotonylation at K390 inhibits mitochondrial membrane potential and aggravates oxidative stress levels in si‐LONP1 Chinese hamster ovary (CHO) cells. (A–D) Empty vector, Flag‐tagged WT LONP1, and LONP1‐K518R, K390R, K350R, and K363R mutant plasmids transfected into si‐LONP1 CHO cells were labeled with MitoTracker Green (A), JC‐1 (B), MitoSOX Red (C), and DCFH‐DA (D) probes and observed under confocal microscope. (E–H) The mean fluorescence intensity of FITC, PE/FITC, and PE in each group were calculated and analyzed. The statistical significance was compared separately to the si‐LONP1 group. All error bars, mean values ± SD, and *p*‐values were determined by unpaired two‐tailed Student's *t*‐test of *n* = 3 independent biological experiments. ^*^ *p* < 0.05; ^**^ *p* < 0.01; ^***^ *p* < 0.001; ^****^ *p* < 0.0001.

As mitochondrial homeostasis is related to LONP1 function and activity, we further examined the mitochondrial function. In LONP1 KD cells with over‐expression of LONP1‐K390R, the fluorescence intensity of MitoTracker Green was weakened, and the mitochondria were sparse and punctate (Figure 5A,E). This indicates that the defects of mitochondria in LONP1 KD cells were not recovered upon LONP1‐K390R over‐expression. In contrast, the fluorescence intensity of MitoTracker Green in LONP1 KD cells was partially rescued upon over‐expression of LONP1 with K518R, K350R, or K363R mutations. In addition, we also evaluated the state of mitochondria by other methods in the LONP1 KD cells with WT or mutated LONP1 over‐expression. JC‐1 was used to verify mitochondrial membrane potential. According to our findings, over‐expression of LONP1‐K390R does not rescue the red fluorescence decrease in LONP1 KD cells, but over‐expression of LOPN1 with the other three types of mutations enables partial rescue (Figure 5B,F). Our findings reflected a notable elevation in MitoSOX Red intensity in LONP1 KD cells that can still be observed upon over‐expression of LONP1‐K390R (Figure 5C,G). Furthermore, intracellular ROS were detected using 2,7‐dichlorodihydrofluorescein diacetate (DCFH‐DA) probe and the changes in DCFH‐DA levels are consistently depicted in Figure 5D,H.

Based on these findings, it was observed that decrotonylation of K390 had a significant impact on the activity of endogenous LONP1 expression, ultimately leading to a disruption of mitochondrial function and an increase in oxidative stress.

### 2.5 LONP1 lysine decrotonylation aggravates mitochondrial dysfunction in vitro

Additionally, we also determined the in vitro LONP1 activities and mitochondrial function with K518R, K390R, K350R, or K363R mutations expressed in the WT CHO cells and HEK‐293t cells.

Detection of intracellular ATP levels based on firefly luciferase catalyzing luciferin to produce fluorescence that requires ATP to provide energy, and the results showed that K390R‐transfected cells showed a decline in ATP levels compared to WT, empty vector (EV), and normal cells. Additionally, the ATP hydrolysis rate of LONP1 protein was evaluated using the malachite green phosphate assay, and the results of phosphate level exhibited a similar trend as that of ATP (Figure S2C), suggesting that LONP1 activity and mitochondrial function were affected by the over‐expression of K390R. Furthermore, quantification was performed via flow cytometry. Notably, the K390R group exhibited a statistically significant reduction in the P‐phycoerythrin (PE)/fluorescein (FITC) ratio of fluorescence intensity in comparison to the WT and blank groups, indicating a diminished mitochondrial membrane potential (*p* < 0.0001) (Figure S2D,E). Consistent with these findings, the results obtained from flow cytometry utilizing the superoxide indicator MitoSOX Red probe also demonstrated a similar trend (Figure S2F,G).

Next, cells were over‐expressed with LONP1‐K518R, K390R, K350R, K363R, WT, and EV plasmids in CHO cells, and were assessed by measuring alterations in oxidative indicators to evaluate the impact of LONP1‐K390 decrotonylation on mitochondrial function. MitoTracker Green was used to measure changes in mitochondrial shape, mass, swelling, size, localization, and structure. The results of confocal microscopy revealed that with the same low concentration of MitoTracker Green probe, the mitochondria of the blank (untreated cells) and WT groups showed fine filamentous and rod‐like morphology, and the distribution was relatively uniform and extensive. However, in the K390R group, the fluorescence intensity of MitoTracker Green was weakened, and the mitochondria were sparse and punctate (Figure S3A,E). The mitochondrial membrane potential was evaluated through the utilization of a mitochondrial probe, JC‐1. Our findings indicate a substantial increase in green fluorescence and a decrease in red fluorescence in the K390R group under confocal microscopy (Figure S3B,F). The level of oxidative stress in vitro was determined by measuring mitochondrial superoxide, the primary ROS within mitochondria, and a byproduct of oxidative phosphorylation. Our results demonstrate a significant increase in MitoSOX Red content in the K390 group (Figure S3C,G). Additionally, intracellular ROS was labeled using a fluorescence probe, DCFH‐DA, and the variation in DCFH‐DA levels is presented consistently, as shown in Figure S3D,H. These results showed that K390R mutation in LONP1 can decrease mitochondrial function compared to WT LONP1 over‐expression, indicating that the K390R mutation plasmid is a dominant tool for modulating LONP1 function or expression as the exogenous forced expression of K390R mutation could compete with WT LONP1 and then attenuate the functions of WT LONP1. The above experiments were also repeated in the HEK‐293t cell line (Figure S4A–H).

### 2.6 LONP1 decrotonylation levels in PCOS oocytes

**FIGURE 6:**
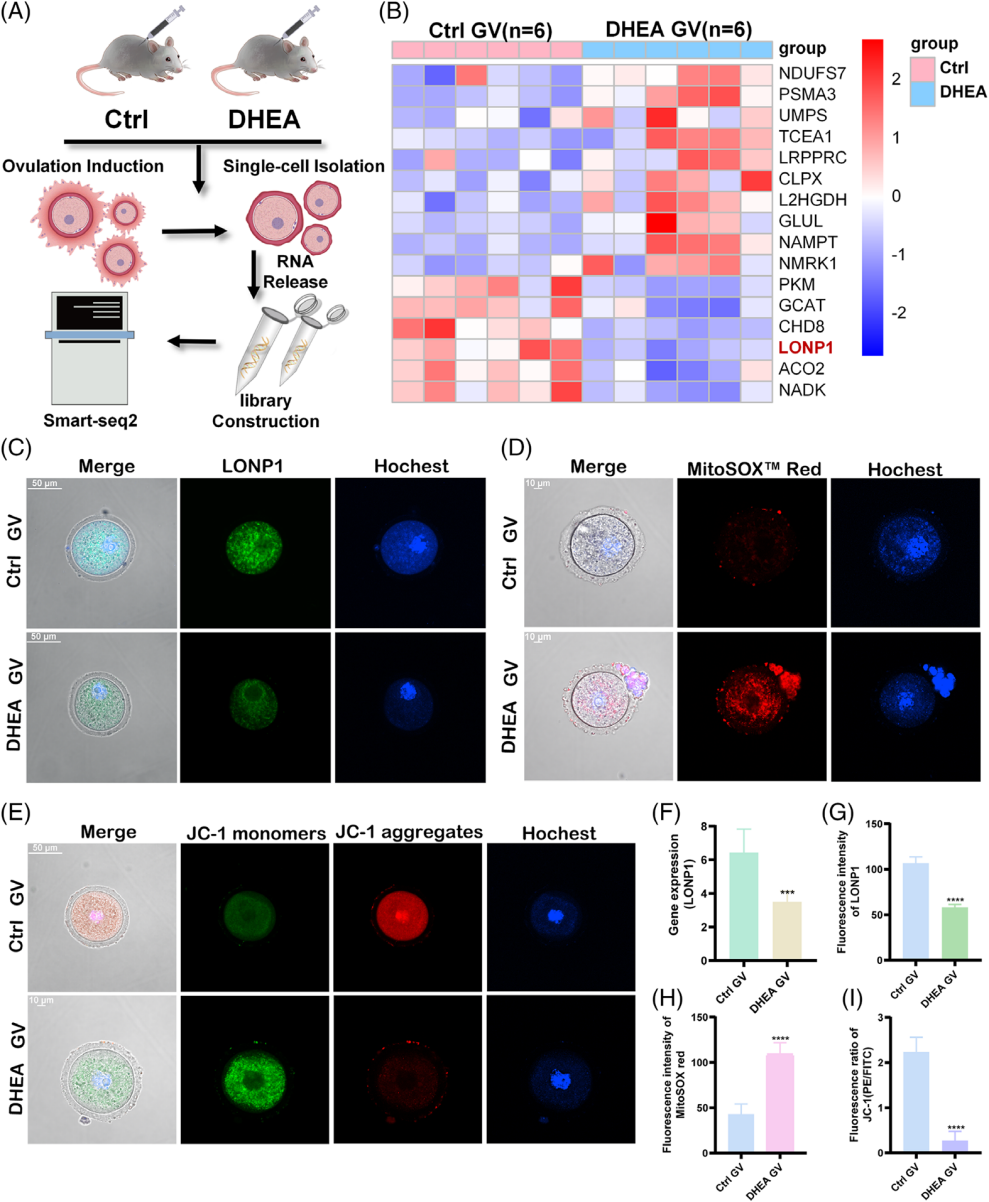
Differential expression of Lon protease 1 (LONP1) in mouse GV stage oocytes aggravates mitochondrial dysfunction in oocytes. (A and B) The flow chart of single‐cell RNA sequencing and mitochondrial‐related gene expression heatmap in mouse GV stage oocytes’ of polycystic ovary syndrome (PCOS)‐like and control groups. The elements used in this flow chart were completed with the assistance of Adobe Illustrator, Adobe Photoshop, and Hefei Sondii Media Co., Ltd. (C, D and E) Observation of LONP1 immunofluorescence (C), MitoSOX Red (D), and JC‐1 (E) fluorescence under confocal microscopy. (F) The LONP1 expression level showed a significant statistical difference between two groups based on single‐cell RNA sequencing results (each group contained six GV stage oocytes). (G, H, and I) The mean fluorescence intensities of FITC (G), PE (H), and PE/FITC (I) in each group were calculated and analyzed. All error bars, mean values ± SD, and *p*‐values were determined by unpaired two‐tailed Student's *t*‐test of *n* = 3 independent biological experiments. ^*^ *p* < 0.05; ^**^ *p* < 0.01; ^***^ *p* < 0.001; ^****^ *p* < 0.0001.

We collected oocytes from the DHEA and control mice, and conducted single‐cell RNA sequencing (scRNA‐seq) (Figure 6A). Raw fastq files were trimmed using TrimGalore‐0.6.5, and we mapped trimmed reads to reference genome mm10 using STAR‐2.7.3a. The quality control results of scRNA‐seq, including number of trimmed reads, average input read length, and uniquely mapped reads, depicted no statistical difference between two groups, which indicated that results met the requirements for single‐cell credit analysis. We focused on the expression of genes related to mitochondrial metabolic pathways that are known to modify ovary crotonylation levels. Then, gene expression heatmap was performed and the correlation between each sample pair was calculated. The expression patterns of the other down‐regulated genes in PCOS mouse germinalvesicle (GV) oocytes are shown in Figure 6B. It was found that LONP1 gene expression was down‐regulated almost a half in PCOS mouse GV stage oocytes (*p* < 0.001) (Figure 6F), which may be related to developmental defects in PCOS oocytes.^23^

To confirm this hypothesis, we observed LONP1 immunofluorescence and employed MitoSOX Red and JC‐1 fluorescence probes under confocal microscopy to examine the degree of oxidative stress and mitochondrial membrane potential in GV stage oocytes. The results showed that the protein levels of LONP1 were notably decreased in DHEA GV oocyte (Figure 6C,G). In addition, MitoSOX Red (Figure 6D,H) and JC‐1 (Figure 6E,I) signals were decreased in DHEA GV oocytes, suggesting that the membrane potential of DHEA GV oocyte was defective. The aforementioned indicators were also detected in oocytes at the MII stage, as reflected in Figure S5A–H.

Next, to investigate how our findings in mice might relate to human PCOS, whole blood samples were collected from 18 women and 18 non‐PCOS controls with comparable ages and body mass indices (Table 1). Women in the PCOS group exhibited a much higher incidence of menstrual cycle irregularities than those in control group. Furthermore, internal secretion and metabolism levels also displayed significant disparities. In the PCOS women, increases in the serum concentrations of T (*p* < 0.001) and an increased ratio of LH to FSH (*p* < 0.01) were observed. In addition, higher homeostasis model assessment of insulin resistance (HOMA‐IR), triglyceride, and low‐density lipoprotein levels (*p* < 0.05) were observed in PCOS patients.

**TABLE 1:** Clinical parameters in polycystic ovary syndrome (PCOS) and normal women.

Parameters	PCOS (*n* = 18)	Normal (*n* = 18)	*p*‐Value
Age (years)	28.3 ± 4.86	29.875 ± 6.53	ns
BMI (kg/m^2^)	22.70 ± 4.05	20.99 ± 1.63	ns
Menstrual cycle	68.75 ± 16.20	28.25 ± 3.20	<0.0001
FSH (IU/L)	5.59 ± 1.6	7.52 ± 1.19	<0.05
LH (IU/L)	9.94 ± 4.31	4.41 ± 1.26	<0.01
LH/FSH	1.89 ± 0.91	0.58 ± 0.14	<0.01
Estradiol (pg/mL)	95.59 ± 42.78	35.5 ± 8.57	<0.01
Progesterone (ng/mL)	0.60 ± 0.69	0.65 ± 0.12	ns
Testosterone (ng/mL)	1.02 ± 0.11	0.47 ± 0.17	<0.001
HOMA‐IR	2.49 ± 2.80	1.04 ± 0.29	<0.05
Total cholesterol (mmol/L)	4.62 ± 0.39	4.32 ± 0.67	ns
Triglyceride (mmol/L)	1.32 ± 0.80	0.66 ± 0.13	<0.05
HDL‐C (mmol/L)	1.25 ± 0.42	1.70 ± 0.58	ns
LDL‐C (mmol/L)	2.79 ± 0.61	2.03 ± 0.42	<0.05

*Note*: *p*‐Values >0.05 were considered non‐significant (ns). HOMA‐IR = [fasting glucose (mmol/L) × fasting insulin (μIU/mL)]/22.5.

Abbreviations: BMI, body mass index; FSH, follicle‐stimulating hormone; HDL, high‐density lipoprotein; HOMA‐IR, homeostasis model assessment of insulin resistance; LDL, low‐density lipoprotein; LH, luteinizing hormone.

**FIGURE 7:**
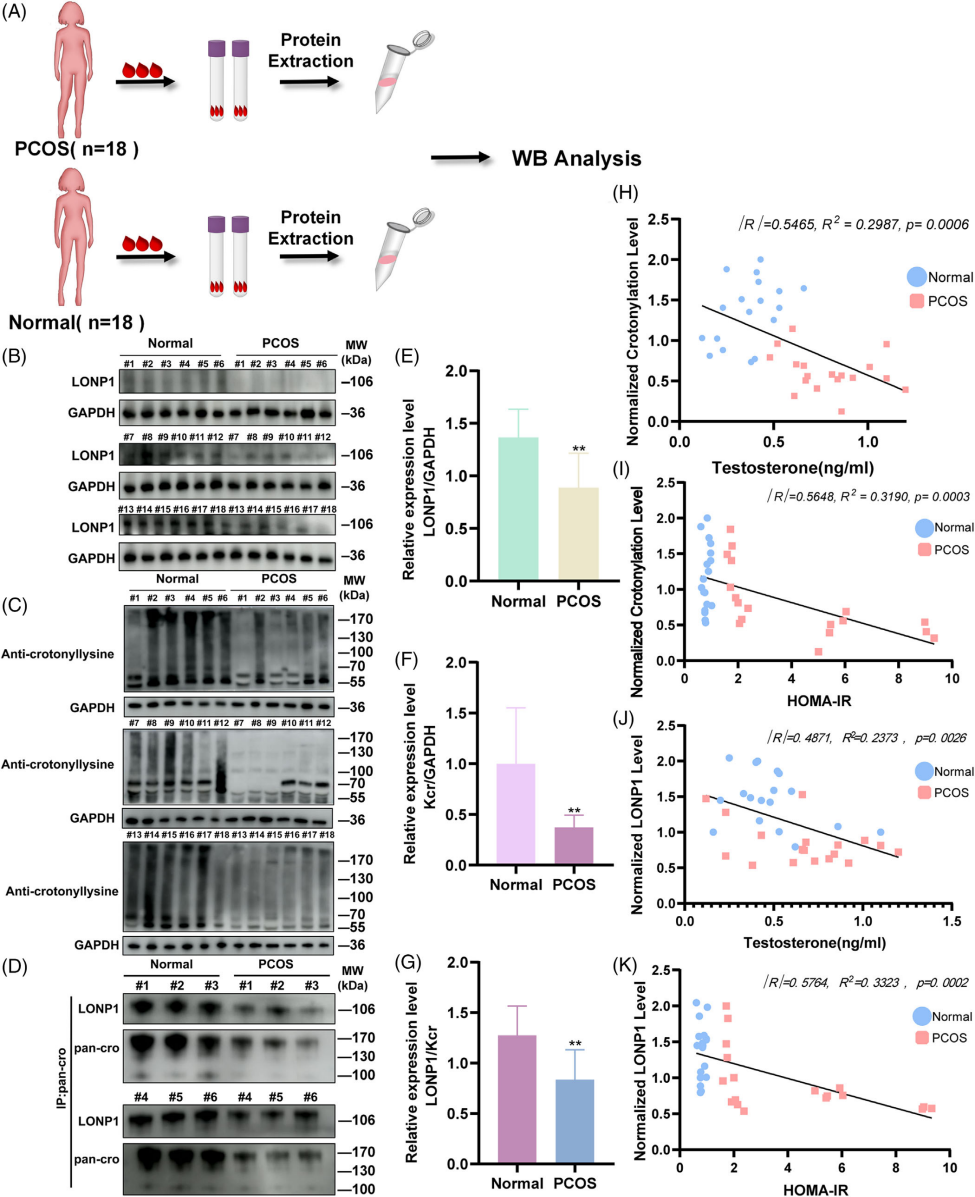
Detection of Lon protease 1 (LONP1) lysine crotonylation in whole blood samples of polycystic ovary syndrome (PCOS) and normal women and its correlation with PCOS clinical phenotypes. (A) Diagram of whole blood collection, lysis, and protein extraction. The elements in the diagram were improved with Adobe Illustrator, Adobe Photoshop, and Hefei Sondii Media Co., Ltd. (B and C) LONP1 and crotonylation levels in whole blood were detected by western blotting with anti‐LONP1 antibody and anti‐pan‐crotonylation antibody, respectively. (D) A co‐immunoprecipitation (Co‐IP) assay was performed with antibody against anti‐pan‐crotonylation antibody to verify the LONP1 crotonylation in PCOS blood samples (*n* = 18 in each group). (E–G) Relative expression levels of LONP1/GAPDH (E), Kcr/GAPDH (F), and LONP1/crotonylation (G). (H and I) The correlation analysis of crotonylation levels with testosterone (H) and homeostasis model assessment of insulin resistance (HOMA‐IR) (I) of PCOS and normal women, *R*: Pearson correlation coefficient. The insulin resistance index (HOMA‐IR) was calculated using homeostasis model assessment methods and was defined as: [fasting glucose (mmol/L) × fasting insulin (μIU/mL)]/22.5. (J and K) The relativity between LONP1 level in blood sample and clinical phenotypes (hyperandrogenism and insulin resistance). All error bars, mean values ± SD, and *p*‐values were determined by unpaired two‐tailed Student's *t*‐test of *n* = 3 independent biological experiments. ^*^ *p* < 0.05; ^**^ *p* < 0.01; ^***^ *p* < 0.001; ^****^ *p* < 0.0001.

The crotonylation, LONP1 expressions, and crotonylation of LONP1 levels in the acquired blood samples were determined (Figure 7A). There was a significant difference between the control and PCOS groups. The expression of LONP1 declined in PCOS group (Figure 7B,E). Similarly, in the PCOS group, the crotonylation level decreased (Figure 7C,F). Furthermore, a Co‐IP assay with antibody against anti‐pan‐cro was performed to verify the LONP1 crotonylation in PCOS blood samples. The crotonylation degree of LONP1 descended, as shown in Figures 7D,G and S6C (*p* < 0.01). Additionally, the WB analysis of LONP1 and pan‐cro by anti‐LONP1 immunoprecipitation (IP) also described in Figure S6A,B with *p* < 0.01.

The correlation between clinical phenotypes and crotonylation levels in the two groups was further analyzed (Table 2). There was a statistically significant association between elevated T concentrations and reduced crotonylation levels (Figure 7H). A similar tendency also occurs in HOMA‐IR, an IR index, and a higher ratio represents greater IR (Figure 7I). LONP1 levels in whole blood protein were also negatively correlated with the clinical phenotype of PCOS (hyperandrogenemia and IR); that is, the lower the LONP1 level, the higher the androgen and IR index (Figure 7J,K). These data suggest that the down‐regulation of LONP1 and crotonylation modification levels are correlated with PCOS phenotype.

**TABLE 2:** The correlation of clinical phenotypes with crotonylation levels of polycystic ovary syndrome (PCOS) and normal women.

Phenotypes	*R* ^2^	*p*‐Value
LH/FSH	0.1849	0.0964
Estradiol (pg/mL)	0.1738	0.1082
Testosterone (ng/mL)	0.2987	0.0006
HOMA‐IR	0.3190	0.0003
Triglyceride (mmol/L)	0.01503	0.6510
LDL‐C (mmol/L)	0.1424	0.1495

*Note*: The whole blood samples from 18 pairs of normal women and PCOS patients were examined to analyze the association of the lysine crotonylation with the clinical phenotypes. The gray value of crotonylation level was normalized with that of GAPDH by Imagel and analyzed by Pearson correlation coefficient test. *p*‐Values <0.05 were considered significant.

Abbreviations: FSH, follicle‐stimulating hormone; HOMA‐IR, homeostasis model assessment of insulin resistance; LDL, low‐density lipoprotein; LH, luteinizing hormone.

## 3 DISCUSSION

PCOS is a highly heterogeneous endocrine disorder, with metabolic disorders and hypo‐reproductive function being the main clinical manifestations.^2^ Researchers have found that the disruption of metabolic homeostasis in the ovary affects ovarian function, including follicular development, maturation, and ovulation.^5^, ^24^ Specifically, the alterations in the intermediate metabolites derived from glycolysis, fatty acid β‐oxidation, branched‐chain amino acid catabolism, TCA cycle, and nicotinamide adenine dinucleotide catabolism in the follicular fluid and cumulus cells of classic PCOS patients lead to mitochondrial dysfunction, imbalanced redox potential, and heightened oxidative stress. The aforementioned changes played a role in impeding of follicular growth and the onset of oocyte developmental anomalies, culminating in a diminished rate of ongoing pregnancies among afflicted females.^24^

The maintenance of metabolic homeostasis is primarily facilitated by mitochondria. Mitochondria are capable of generating intermediates for biosynthesis and possess a dual effect on the intracellular redox potential^25^; they are also the primary source of cellular ROS. However, the overproduction of oxidative ROS leads to the denaturation of mitochondrial matrix proteins, resulting in mutations, abnormal expression, or misfolding. Maintaining mitochondrial quality control to preserve cellular homeostasis and prevent cellular damage is crucial. LONP1, a highly conserved serine peptidase, plays a critical role in the protein quality control system in mammalian mitochondria.^26^ As a member of the AAA+ (ATPases associated with various cellular activities) family of proteins, LONP1 requires ATP hydrolysis to degrade proteins. LONP1 is responsible for catalyzing the degradation of oxidized, dysfunctional, and misfolded matrix proteins within the mitochondria while also regulating mitochondrial gene expression and genome integrity. This multifunctional protein exhibits proteolytic, molecular chaperone, and mtDNA binding activities, and is involved in regulating various cellular processes both within and outside the mitochondria.^27^ Its proteolytic activity plays a crucial role in regulating several biochemical pathways, including the TCA cycle and oxidative phosphorylation, steroid and heme biosynthesis, and glutamine biosynthesis.^9^, ^28^ According to a recent study, a reduction in proteolytic activity of LONP1 was observed in aging rat liver cells,^29^ resulting in the accumulation of damaged proteins. Notably, the protein level of LONP1 remained unchanged, indicating the possible involvement of protein PTMs.

PTMs, such as phosphorylation, acetylation, glycosylation, methylation, etc., play a significant role in various cellular activities, including signal transduction, regulation of protein stability and activity, gene expression, and genome integrity maintenance. Among these modifications, lysine crotonylation has garnered considerable attention as a novel form of histone PTM.^30^ The lysine crotonylation of non‐histone proteins that was first discovered in 2017 was also widespread.^31^ Kcr is a key to regulate cellular functions in both physiological and pathological states, such as DNA damage^32^ and repair,^33^ gametogenesis,^34^ sperm motility,^35^ endoderm differentiation,^36^osteogenic differentiation,^37^ neural development,^12^ pancreatic cancer progression,^38^ hepatocellular carcinoma progression,^39^ cardiac homeostasis,^40^ acute kidney injury,^13^ diabetic kidney disease,^41^ hepatic metabolism,^15^ glucose metabolism,^42^ and depression.^43^ In PCOS, the acetylation and methylation^44^ of histones^45^ and non‐histones^5^ have been reported in many studies, but crotonylation has not been reported yet.

In this study, we first performed a comprehensive in vitro analysis of ovarian Kcr modification in PCOS mice and investigated the significance of mitochondrial damage in PCOS using several representative site‐specific mutants. LONP1 is a mitochondrial protease essential for maintaining mitochondrial protein balance and alleviating cellular stress,^46^ and it has been shown that mitochondrial LONP1 protects cardiomyocytes from ischemia‐reperfusion injury in vivo.^47^ Another study described that LONP1 plays a principal role in the proteolysis of non‐specific 5‐amino levulinate synthase (ALAS1) in the mitochondria. Intracellular expression of ALAS1 is a vital enzyme that regulates the production of heme or heme precursors.^48^ Excessive heme or heme precursors lead to the production of ROS, which aggravate oxidative stress in cells. In addition, skeletal muscle‐specific LONP1 ablation in mice causes impaired mitochondrial protein turnover, leading to mitochondrial dysfunction, thus aggravating decreased skeletal muscle mass and strength.^49^ These studies revealed that LONP1 plays a significant role in regulating mitochondrial homeostasis and that its activity is associated with metabolism‐related dysfunction.^50^, ^51^

Our study proposes that the mitochondrial protein LONP1 may regulate metabolic homeostasis in the PCOS ovarian micro‐environment through Kcr. We have substantiated this hypothesis through two distinct approaches. First, we verified the direct link between K390 crotonylation and the physiological function of endogenous LONP1 by using KD LONP1 CHO cells. Compared to other three sites, the over‐expression of K390 mutant crotonylation site could not rescue the mitochondrial dysfunction caused by the KD of LONP1. Decrotonylation of LONP1‐K390 suggests that regulating the specific Kcr sites of specific proteins, disrupts metabolic homeostasis in the ovarian micro‐environment by inhibiting LONP1‐mediated mitochondrial function, including the quality of oocytes, as evidenced by the observed reduction in mitochondrial transmembrane potential and elevation of MitoSOX in GV and MII oocytes of PCOS‐like mice. Also, we demonstrated that exogenous forced expression of 390 site‐mutated LONP1 may competitively inhibit the function of WT LONP1, which led to the disruption in mitochondrial function. This supported that K390R mutation plasmid is a dominant tool for modulating LONP1 function and expression. Previous reports have postulated hypotheses concerning competitive inhibition of this condition. Carvill et al.^52^ proposed that this pathogenic mechanism arises when some property of the mutant protein interferes with the function of the WT protein that is translated from the unaffected allele.

Previous studies have demonstrated a correlation between high androgen levels and mitochondrial dysfunction in PCOS, which can induce mitochondrial dysfunction in vitro and cause elevated OS and pancreatic cell failure in an androgen receptor‐dependent manner.^53^, ^54^ Hyperandrogenism but not obesity induced changes in oxidative stress metabolites in the follicular fluid and cumulus cells of classic PCOS patients.^24^ High androgen levels cause a high OCR, altered ATP production, and reduction in mtDNA copy number within PCOS mouse oocytes. In this study, we found that LONP1 Kcr was significantly down‐regulated in the peripheral blood of PCOS patients with hyperandrogenism. This suggests that lysine crotonylation is a regulatory factor that alters gene and protein expression, contributing to PCOS phenotypic manifestations. These findings provide a novel mechanism for the cause of PCOS, while also identifying ways to improve the ovarian micro‐environment of PCOS.

The main limitation of our study is that we could not identify the specific mechanism that caused the decrease in global crotonylation in PCOS ovarian tissues and peripheral blood and that the causal relationship between them is not clearly defined. We speculate that decrotonylation of mitochondrial proteins is one of the main causes of this phenomenon, possibly through the down‐regulation of LONP1 crotonylation, which affects its activity and function, thus aggravating mitochondrial dysfunction and leading to energy metabolism disorders in the ovary. Further exploration of the molecular mechanisms by which LONP1 regulates mitochondrial function through down‐regulation of crotonylation will be focused.

In this study, the first high‐resolution MS‐based assessment of differential Kcr in ovarian tissues in PCOS mice was reported. We discovered a novel down‐regulation of crotonylation phenomenon in PCOS, both in peripheral blood and ovarian tissues. Protein Kcr participates in various biological processes in PCOS and plays an important role in the regulation of metabolic processes. Modification, through decrotonylation, of the key ATP protease, LONP1, may affect cellular mitochondrial function, and metabolic homeostasis of ovarian epithelial cells and the ovarian microenvironment, which provides new insights into the pathogenesis of PCOS.

## 4 METHODS AND MATERIALS

### 4.1 LONP1 KD with siRNA and shRNA and plasmid preparation

Endogenous LONP1 was KD using correspondingly targeted siRNA (c‐149012) and control siRNA (sc‐37007), both purchased from Santa Cruz. Stable KD of endogenous LONP1 was achieved using lentiviral vector harboring shRNA, which was first transfected into CHO cells, and then selected by puromycin of the infected cells.

The WT Lon protease homolog (LONP1) over‐expressing plasmid and EV plasmid were constructed by amplifying the corresponding sequences and ligating them into pcDNA3.1‐3xFlag‐C vectors. Decrotonylated LONP1 mutants (K518R, K390R, K350R, and K363R) were generated by site‐directed mutagenesis based on Lonp1‐WT NP_083058.2. The WT and four site‐mutant plasmids contained synonymous mutation sequences related to siRNA and shRNA to avoid the KD effect of siRNA and shRNA targeting LONP1. The specific sequences of LONP1 siRNA and shRNA, and synonymous mutations of LONP1 plasmids are shown in Table **S1**. All the sequences were verified by polymerase chain reaction amplification.

### 4.2 LONP1 activity assay

The ATP hydrolysis rate of LONP1 protein was assessed using the malachite green phosphate assay kit (MAK307, Sigma). Forty microliters of 1 mM phosphate standard solution was transferred into 960 L of ultrapure water to prepare a 1 mL premix containing 40 μM phosphate (a phosphate standard solvent). Phosphate standard and test samples (80 μL each) were added into separate plate wells, which contained 20 μL of working solution (100 volumes of reagent A and 1 volume of reagent B). The plate was incubated at 20°C−25°C for 30 min, and the OD of the samples was measured at 620 nm with a microplate reader (BioTek, Synergy H1). All assays were performed in accordance with the manufacturer's instructions.

### 4.3 Evaluation of ATP content

Firefly luciferase, was used to analyze ATP concentration. At a certain concentration range, fluorescence production was proportional to the concentration of ATP. The transfected cells were covered with lysis buffer provided by the ATP assay kit (S0026, Beyotime) and centrifuged at 12,000 × *g* for 5 min (4°C). The supernatant was collected and used for subsequent assays. All samples were kept on ice to reduce enzymatic ATP hydrolysis. ATP concentration was measured using a luminometer (BioTek, Synergy H1).

### 4.4 Seahorse assay

An XFe96 flux analyzer (Seahorse Biosciences, Agilent) was used to analyze the mitochondrial OCR. Briefly, 5 × 10^3^ cells were plated into XF‐96 cell culture microplates in 80 μL of Dulbecco's modified Eagle medium and incubated at 37°C overnight. Cell mitochondria stress test was conducted by adding oligomycin (1.5 μM), trifluoromethoxy carbonylcyanide phenylhydrazone (1.0 μM), and rotenone/antimycin A (both 0.5 μM). Hoechst was used for nuclear staining. OCR values were normalized by cell counting (Cytation 7, Cell Imaging Multi‐Mode Reader, Agilent BioTek).

### 4.5 Mitochondrial distribution labeled by MitoTracker Green

MitoTracker Green is a green fluorescent probe that can be used for mitochondria‐specific fluorescence in living cells. The transfected cells were incubated in a pre‐warmed 37°C staining solution containing 200 nM MitoTracker Green probe (C1048, Beyotime) for 30 min. Following incubation, the samples were washed three times to remove the residual probe. Next, cells were stained with 1× Hoechst staining solution for live cells (C1027, Beyotime) for 10 min, which binds to the cell nucleus, and rinsed with medium droplets three times. The cells were observed to evaluate the mitochondrial distribution using a confocal microscope.

### 4.6 ROS and MitoSOX detection

The predominant ROS in mitochondria is mainly reflected by mitochondrial superoxide, which is readily revealed by the MitoSOX Red reagent. The transfected cells were covered in 1 mL (5 μM) MitoSOX reagent working solution (M36008, Invitrogen) for 10 min at 37°C in the dark and immediately observed under a confocal microscope (Nikon, AXR).

Intracellular ROS were labeled using the fluorescence probe DCFH‐DA (S0033S, Beyotime). The transfected cells were incubated with 10 μM diluted DCFH‐DA probe for 30 min at 37°C in the dark. The cells were evaluated using a confocal microscope after rinsing with the medium three times.

### 4.7 Mitochondrial membrane potential assay with JC‐1

The mitochondrial membrane potential (Δ*Ψ*_m_) was quantified using JC‐1. The decrease in mitochondrial membrane potential, that is, the transition of JC‐1 from red (J‐aggregates) to green (monomers) fluorescence, is a hallmark event in the early stage of apoptosis. After treatment with 1 mL diluted JC‐1 working reagent at 37°C for 20 min in an incubator (C2006, Beyotime), the transfected cells were transferred to fresh medium after washing three times with 1× JC‐1 staining buffer. Finally, the distribution of JC‐1 was evaluated using confocal microscopy.

### 4.8 Statistical analysis

The data are presented as the mean ± SD and were analyzed utilizing GraphPad Prism software (version 8.0.1.244; GraphPad Software Inc.). Statistical significance was evaluated through one‐way analysis of variance using IBM SPSS Statistics software (version 22.0). Differences between groups were assessed using a two‐tailed unpaired Student's *t*‐test, one‐way analysis of variance (ANOVA), or two‐way ANOVA, depending on the specific conditions. The results were deemed statistically significant at *p* < 0.05. Each experiment was replicated at least three times.

The remaining detailed methods are provided in the Supporting Information of this paper.

## AUTHOR CONTRIBUTIONS

*Software, data curation, formal analysis, and visualization*: Y.X. and Q.X. *Writing—original draft preparation and review and editing*: Y.X. and Q.X. *Clinical sample collections and animal experiments*: S.W.C., Y.T., Z.X.G., X.Y.H., and P.H.F. *Conceptualization and design, administration, and funding acquisition*: Q.Y. and Q.X. All authors have read and approved the final manuscript.

## CONFLICT OF INTEREST STATEMENT

The authors declare they have no conflicts of interest.

## ETHICS STATEMENT

All animal procedures were approved by the Committee on the Ethics of Animal Experiments of the Peking Union Medical College Hospital (XHDW‐2022‐037). The parts involving the use of clinical samples were approved by the Research Ethics Board of Peking Union Medical College Hospital (JS‐3213) and were performed in accordance with the World Medical Association Declaration of Helsinki. Written informed consent was obtained from all of the participants.

## Supporting information

Supporting Information

## Data Availability

The data that support the findings of this study are available from the corresponding author (Qi Yu) upon reasonable request.
