# Supplementary material for: Down‐regulation of Lon protease 1 lysine crotonylation aggravates mitochondrial dysfunction in polycystic ovary syndrome
Source: MedComm (2020). 2023 Oct 9;4(5):e396. doi: 10.1002/mco2.396 (PMC10560969; doi:10.1002/mco2.396)
Supplement: Supplementary file 1 — Supporting Information [file MCO2-4-e396-s001.docx]

**Supplementary information**

***Down-Regulation of Lon Protease 1 Lysine Crotonylation Aggravates Mitochondrial Dysfunction in Polycystic Ovary Syndrome***

*Short title: LONP1 decrotonylation aggravates PCOS mitochondria dysfunction*

Yuan Xie^1^, Shuwen Chen^1^, Zaixin Guo^1^, Ying Tian^1^, Xinyu Hong^1^, Penghui Feng^1^, Qiu Xie^2^*, Qi Yu^1^*

^1^Department of Obstetrics and Gynecology, National Clinical Research Center for Obstetric & Gynecologic Diseases, State Key Laboratory for Complex Severe and Rare Diseases, Peking Union Medical College Hospital, Chinese Academy of Medical Sciences & Peking Union Medical College, Peking Union Medical College Hospital (Dongdan Campus), No.1 Shuaifuyuan Wangfujing Dongcheng District, Beijing, 100730, China.

^2^Department of Medical Research Center, State Key Laboratory for Complex Severe and Rare Diseases, Peking Union Medical College Hospital, Chinese Academy of Medical Science and Peking Union Medical College, Beijing 100730, China.

**^*^Correspondence:**

Qi Yu

yuqi2008001@sina.com

Qiu Xie

xieqiu1999@126.com

**1 Supplementary Figures, Legends and Table**

**
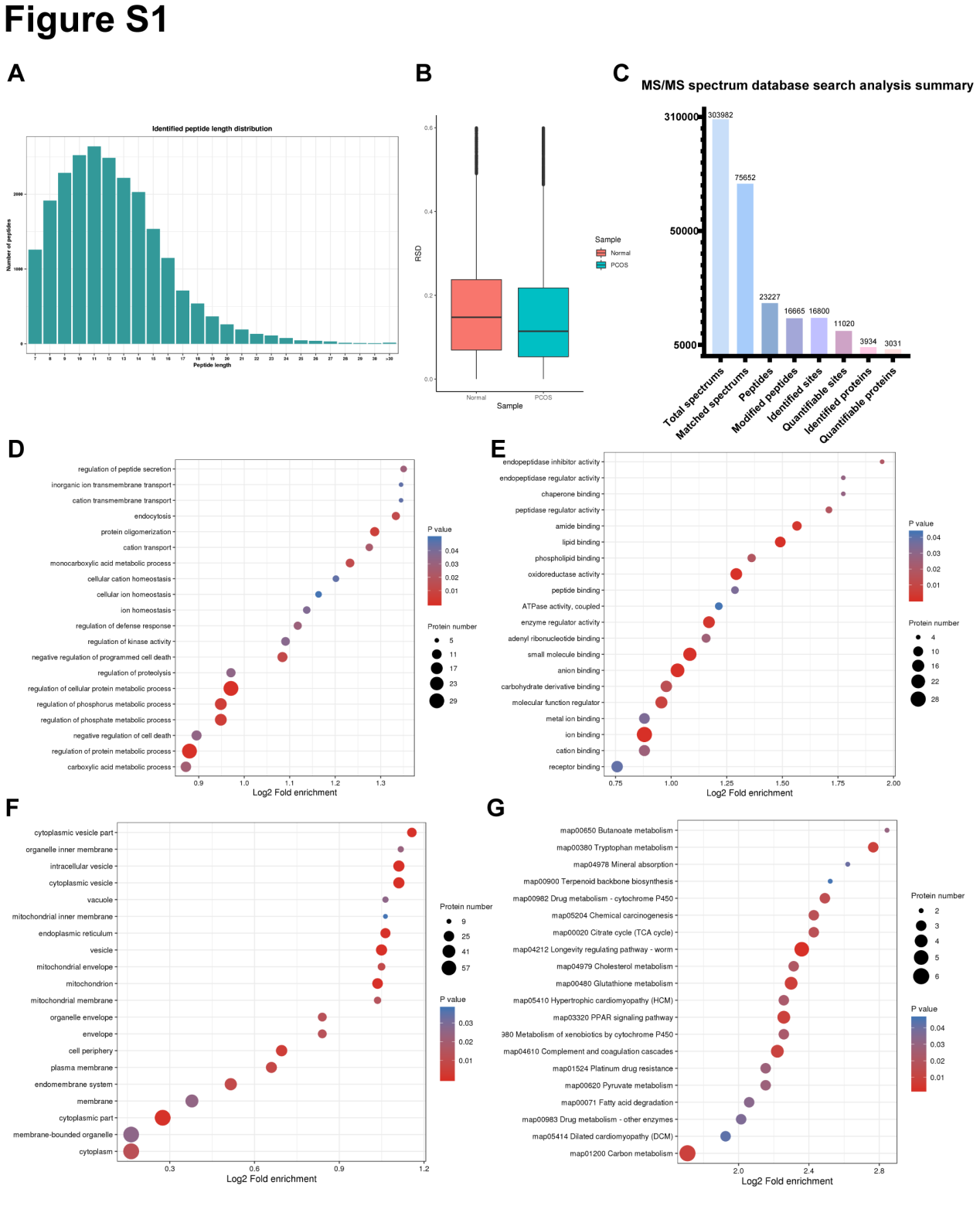
Figure S1 | The QC validation of the MS data. Functional annotation and GO enrichment analysis of differentially crotonylated proteins. (A)** Peptide length distribution. (**B)** relative standard deviation of samples. (**C)** Bar chart showed basic statistical map of the mass spectrometry data results. **(D)** Biological process analysis **(E)** Molecular function analysis **(F)** Cellular component analysis of differentially crotonylated proteins in PCOS and normal groups were enriched by GO term. **(G)** KEGG pathway enrichment of proteins with significant change of lysine crotonylation.

**
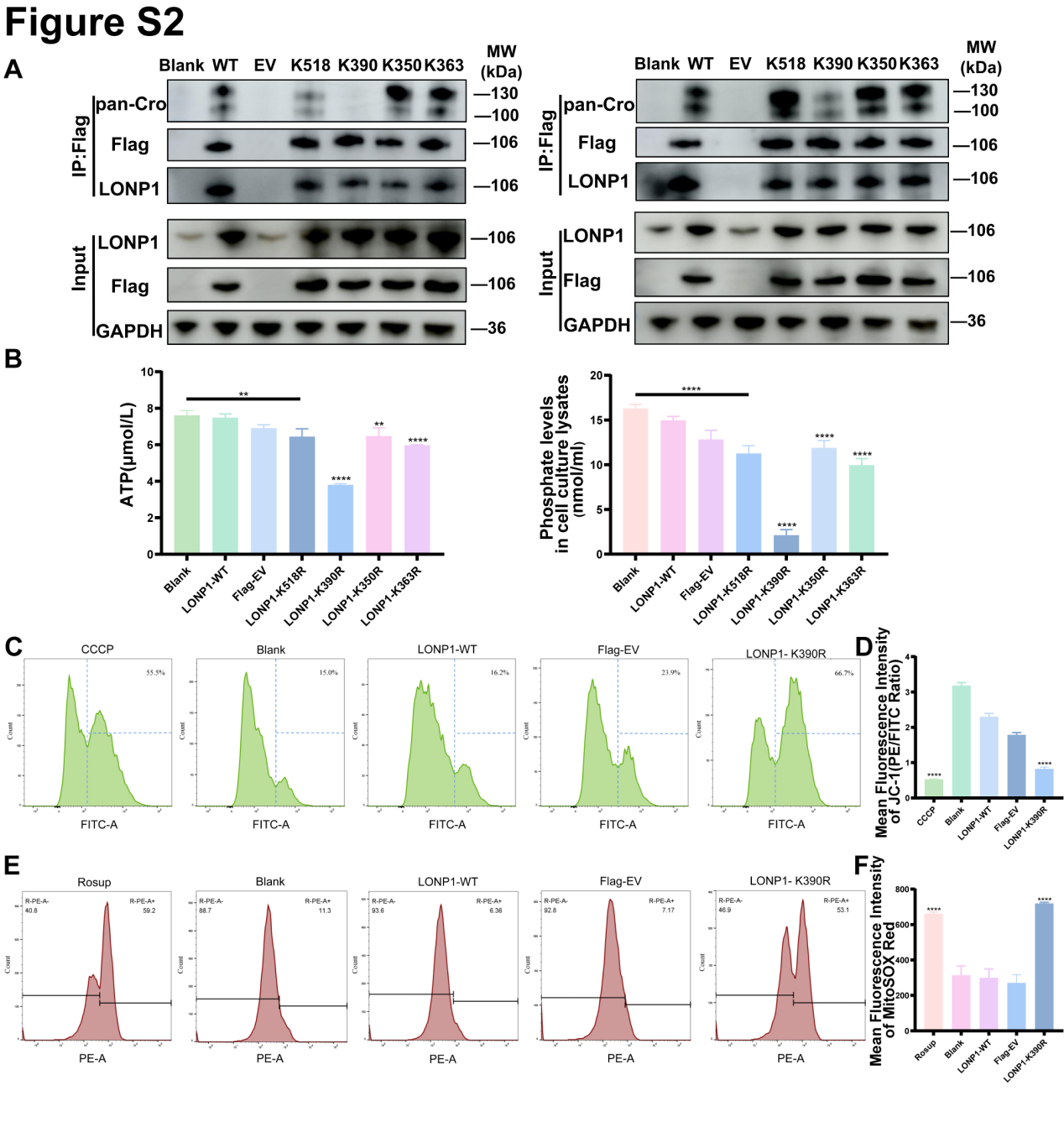
Figure S2 | Down-regulation of LONP1 lysine crotonylation at K390 inhibits ATP hydrolysis rate, mitochondrial membrane potential and aggravates oxidative stress level in CHO cells. (A)** The crotonylation level of LONP1 in Empty vector, Flag-tagged WT LONP1 and the K518R, K390R, K350R, K363R mutant CHO cells. The figure shows the WB image of two additional independent experiments in Figure 3B. **(B)** Green fluorescence observation of GFP-control and GFP-LONP1 shRNA transfecting into CHO cells after 48 h following transfection. (**C)** Detection of intracellular ATP levels of CHO. The rates of ATP hydrolysis of the LONP1 forms were assessed using the Malachite Green Phosphate assay. **(D, E)** The ratio of J-aggregates and monomers in CHO cells was detected by flow cytometry, and the average fluorescence intensity of PE/FITC in each group were calculated. **(F, G)** Use flow cytometry to detect the proportion of Mitosox^TM^ red in CHO cells, and calculate the mean fluorescence intensity of PE in each group. (All error bars, mean values ± SD, *p* values were determined by unpaired two-tailed Student’s t test of n = 3 independent biological experiments. **p* < 0.05; ***p* < 0.01; ****p* < 0.001; *****p* < 0.0001).

**
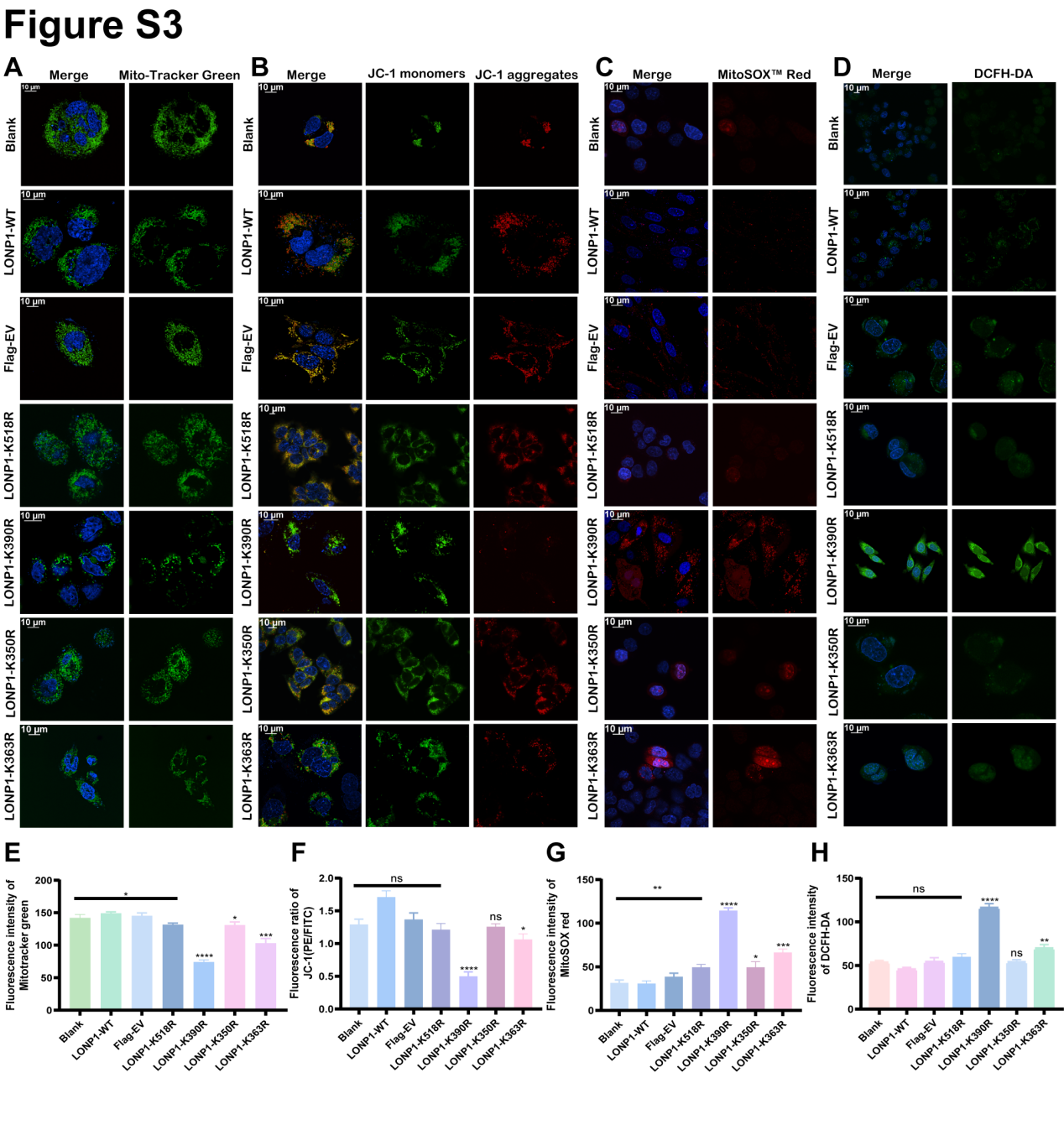
**

**FIGURE S3 | Down-regulation of LONP1 lysine crotonylation at K390 attenuated mitochondrial function in CHO cells. (A-H)** Untreated cells(Blank), Empty vector (EV), Flag-tagged WT LONP1(WT) and the K518R, K390R, K350R, K363R mutant plasmids transfected into CHO cells were labeled with Mito-Tracker Green **(A, E)**, JC-1 **(B, F)**, Mitosox^TM^ red **(C, G)** DCFH-DA **(D, H)** probes and observed under confocal microscope. The statistic significance was compared separately to the Blank group. (All error bars, mean values ± SD, *p* values were determined by unpaired two-tailed Student’s t test of n = 3 independent biological experiments. **p* < 0.05; ***p* < 0.01; ****p* < 0.001; *****p* < 0.0001).

**
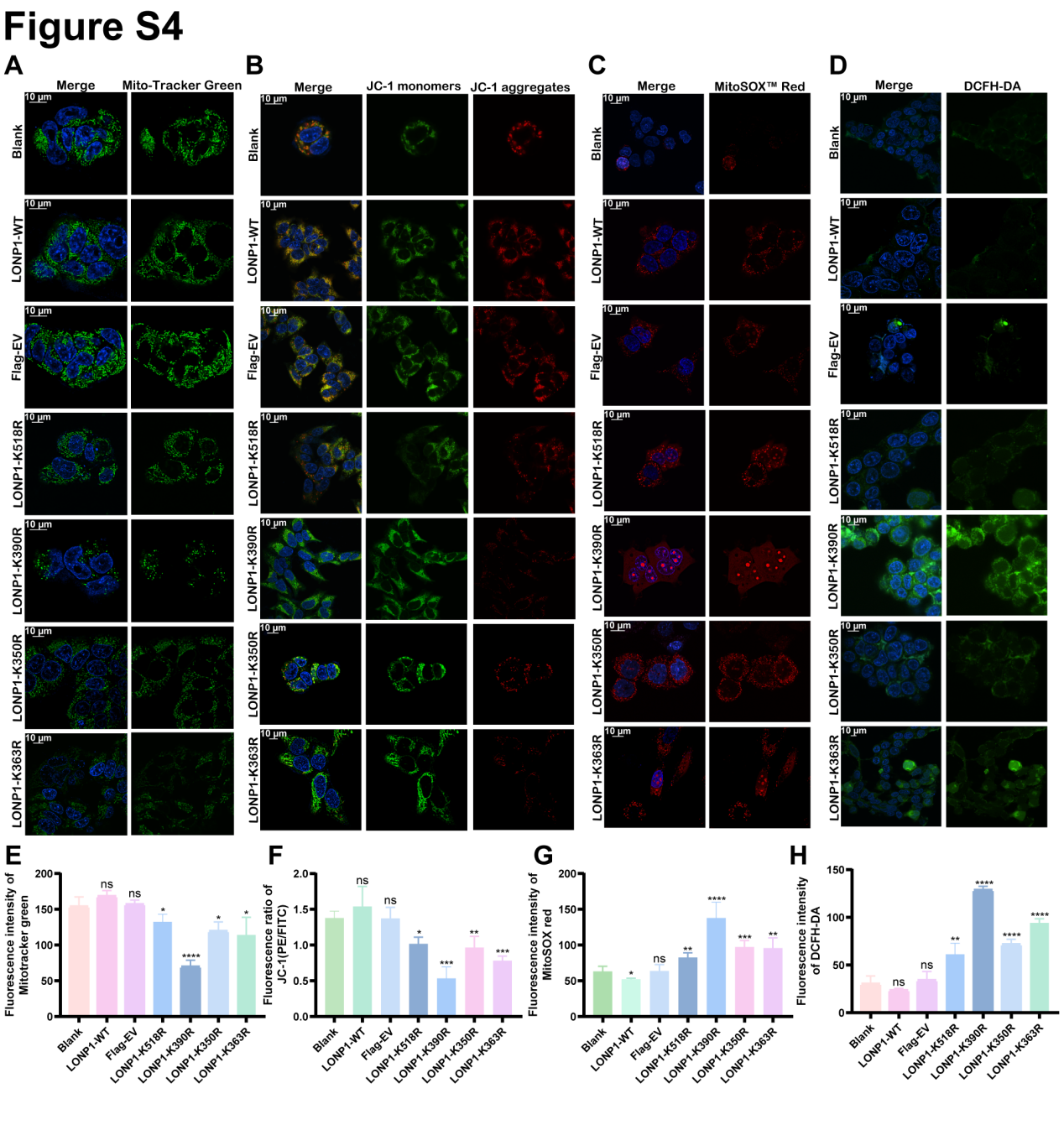
Figure S4 | Down-regulation of LONP1 lysine crotonylation at K390R aggravates mitochondrial dysfunction in HEK-293T cells. (A-D)** Empty vector, Flag-tagged WT LONP1, K518R, K390R, K350R and K363R mutant plasmids transfected into HEK-293T cells were labeled with Mito-Tracker Green **(A),** JC-1**(B)**, Mitosox^TM^ Red **(C)** and DCFH-DA**(D)** probes and observed under confocal microscope. **(E-H)** The mean fluorescence intensity of FITC , PE/FITC and PE in each group were calculated and analyzed. The statistic significance was compared separately to the Blank group (untreated cells) and K390R group. (All error bars, mean values ± SD, *p* values were determined by unpaired two-tailed Student’s t test of n = 3 independent biological experiments. **p* < 0.05; ***p* < 0.01; ****p* < 0.001; *****p* < 0.0001).

**
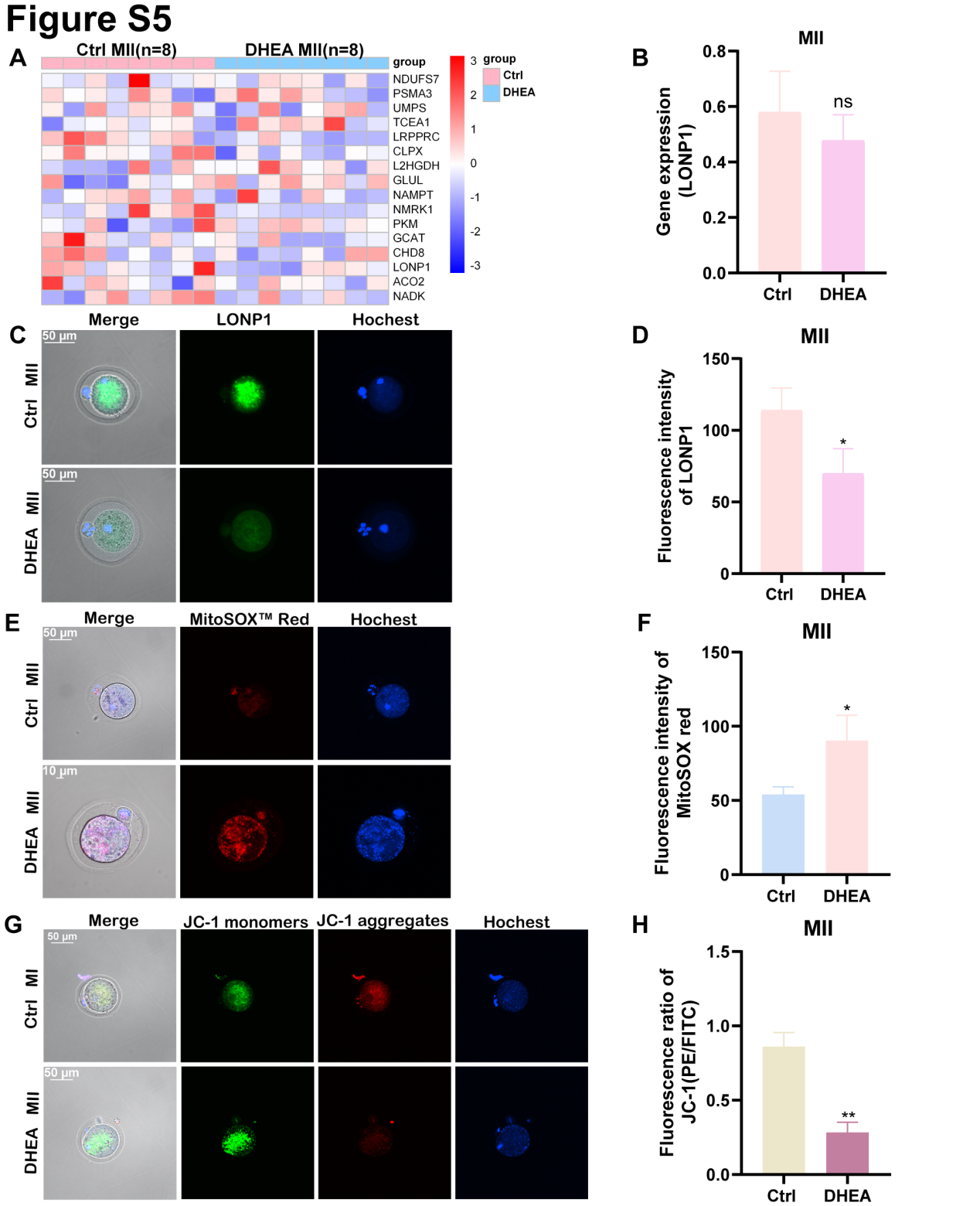
Figure S5 | Gene expression level of LONP1 and mitochondrial function detection in MII stage oocytes from PCOS-like and control mice. (A)** Mitochondrial related gene expression heat-map in mouse MII stage oocytes’ of PCOS-like and control groups. **(B)** LONP1 expression level shown no significant statistical difference in two groups based on single-cell RNA sequencing results (each group contained 8 MII stage oocytes) **(C, E and G)** Observation of LONP1 immunofluorescence **(C)**, MitoSOX^TM^ Red **(E)**, and JC-1 **(G)** fluorescence under confocal microscopy. The mean fluorescence intensity of FITC **(D)** , PE **(F)** and PE/FITC **(H)** in each group were calculated and analyzed. (All error bars, mean values ± SD, *p* values were determined by unpaired two-tailed Student’s t test of n = 3 independent biological experiments. **p* < 0.05; ***p* < 0.01; ****p* < 0.001; *****p* < 0.0001).

**
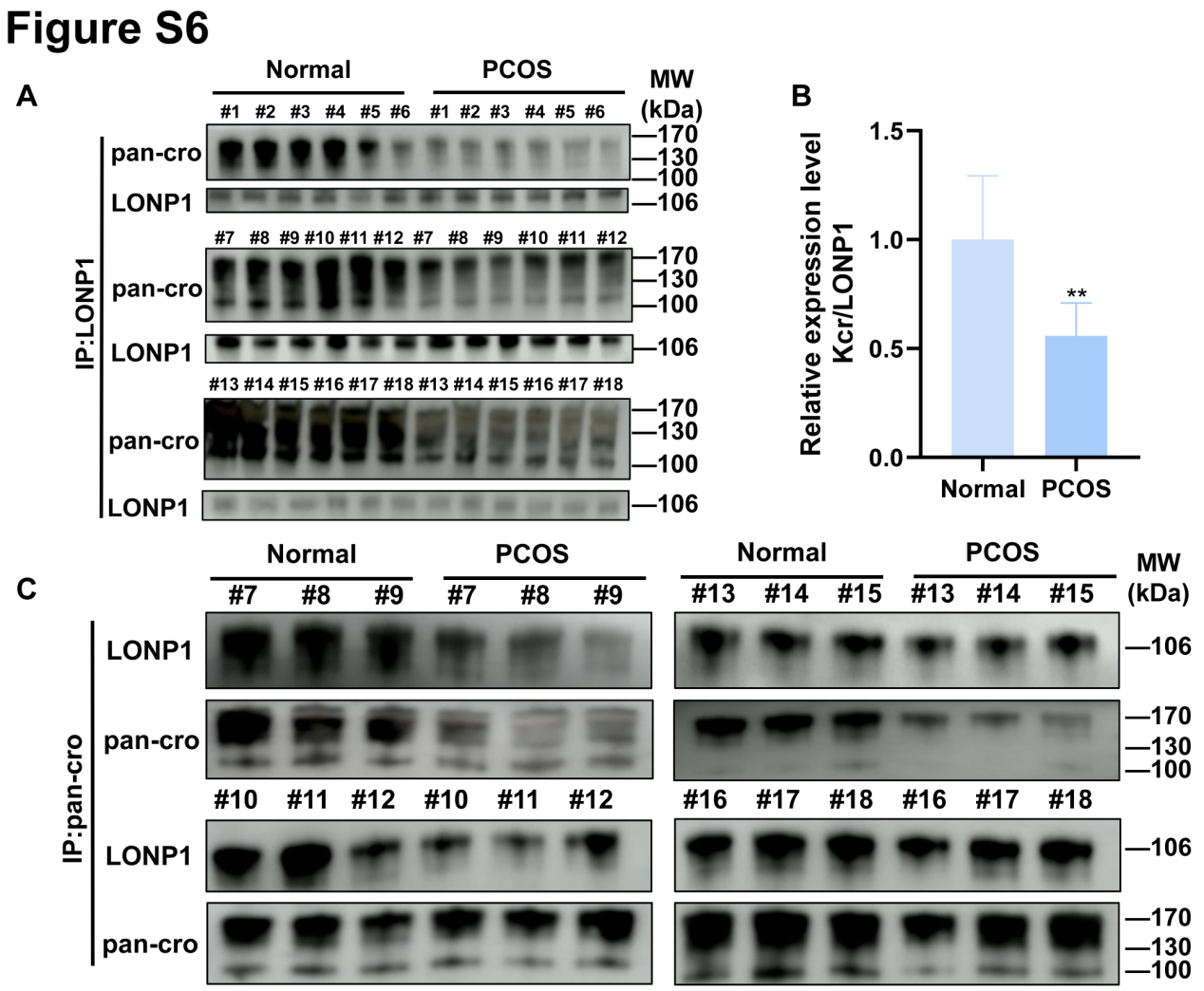
Figure S6 | Detection of lysine crotonylation and LONP1 expression in whole blood of PCOS and normal groups. (A, B)** Co-IP assay between LONP1 and kcr. The whole blood protein obtained from 18 PCOS and 18 normal women were immunoprecipitated with the LONP1-coupled beads, then the expression of crotonylation was observed in WB **(A)** and calculated the significance of statistical **(B)**. **(C)** Western blot analysis of LONP1 protein from PCOS and normal women against anti-pan-crotonylation antibody. This diagram shows the WB images that is not exhibited in the Figure 7D. (All error bars, mean values ± SD, *p* values were determined by unpaired two-tailed Student’s t test of n = 3 independent biological experiments. **p* < 0.05; ***p* < 0.01; ****p* < 0.001; *****p* < 0.0001).

**Table S1. Sequences of LONP1 siRNA and shRNA, and synonymous mutations of LONP1 plasmids**

| **ID** | **Sequence information** | **Codon** | **Synonymous mutation site (highlighted in yellow)** |
| --- | --- | --- | --- |
| sh-LONP1 | cctgagaactagactt | cct | ccg |
| si-LONP1-1 | cgctcagattcatgagatg | gct | gcc |
| si-LONP1-2 | gaagaccattcgggatatc | aag | aaa |
| si-LONP1-3 | ggaacactatcgggacata | gaa | gag |

**2 Supplementary Methods and Materials**

**2.1 Study Design and Animal Experiments**

C57BL/6N female mice were obtained, at three weeks old, from the Beijing Vital River Laboratory Animal Technology Corporation, and experiments were performed in accordance with the National Institutes of Health’s Guide for the Care and Use of Laboratory Animals. The animals were acclimatized for one week, before initiating experiments, and maintained under specific pathogen-free conditions. Subsequently, the mice were injected for 21 consecutive days, with 6 mg/100 g body weight (BW) dehydroepiandrosterone (DHEA) (D4000; Sigma-Aldrich, St. Louis, MO, USA) per 0.05 mL of sesame oil, to induce hyperandrogenic PCOS. Control mice were injected with 0.05 mL of sesame oil^22^. The mice were maintained in a temperature-controlled environment with free access to food and water under a standard dark-light cycle. Mice were group-housed with up to six mice per cage. All procedures were approved by the Committee on the Ethics of Animal Experiments of the Peking Union Medical College Hospital（XHDW-2022-037）.

**2.2 Estrous cycle monitoring**

During the molding period, vaginal smear was used to determine the mice estrous cycle. An appropriate amount of physiological saline (10–20 µL) was injected into the mouse vagina. A swab was performed and the collected cells were transferred onto a dry glass slide. The slide was dried naturally, and cells were fixed with methanol for 3 min^22^. After fixing, the mouse vaginal cell morphology was observed under a microscope. The estrous cycle was categorized into three phases: the early, late, and intergenic phases.

**2.3 Histological assessment of mouse ovaries**

Dissected ovaries were weighed and fixed in 4% paraformaldehyde overnight at 4°C. After dehydration, ovaries were embedded in paraffin, serially sectioned into 5 µm slices. The excised ovaries were fixed using 4% paraformaldehyde and stained with hematoxylin and eosin (H&E)^22^. The stained ovaries were examined using Nano Zoomer Digital Pathology RS.

**2.4 Measurement of mice response to insulin and glucose**

Insulin tolerance tests (ITTs) and glucose tolerance tests (GTTs) were performed on day 18 of modeling as previously described^22^.

**2.5 Serum analysis of mouse sex hormones**

The levels of sex hormones, contained in serum mice samples, were by quantified by ELISA using the luteinizing hormone (JM-02865M1), follicle stimulating hormone (JM-02838M1), and testosterone (JM-02852M1) ELISA kits (Jingmei Biotechnology; Yancheng, Jiangsu, China) according to manufacturer’s indications. The optical densities were read at 450nm using the modulus microplate multimode reader (Turner Biosystems, New York, USA)

**2.6 Gene expression pattern analysis of PCOS-like mice oocytes**

**Oocyte collection.** Superovulation was induced as previously described^56^. Briefly, mice were injected, intraperitoneally, with w10 IU pregnant mare serum gonadotropin (PMSG; Ningbo Second Hormone Factory) and 10 IU human chorionic gonadotropin (hCG; Ningbo Second Hormone Factory). GV oocytes were collected 48h after PMSG and MII Oocytes were harvested 24h after hCG administration. The oocytes were released into pre-heated G-MOPS^plus^ medium (Vitrolife, Sweden). Cumulus-free oocytes were released from the oocyte-corona-cumulus complex after treatment with 0.3 mg/mL hyaluronidase (Sigma-Aldrich), and incubated at 37°C in G-IVF^plus^ medium (Vitrolife, Sweden) for subsequent experiments.

**cRNA-seq data processing.** Sequencing reads were trimmed using Trim Galore (version 0.6.5), which is a Perl wrapper based on two tools: Cutadapt and FastQC. For mouse scRNA-seq data, reads with quality > 20 were aligned to the mouse reference genome (mm10) using STAR (version 2.7.3a)49. For human scRNA-seq data, reads with quality > 20 were aligned to the human reference genome (hg38) using STAR (version 2.7.3a). Low mapping quality sequencing reads were removed using Samtools version 1.7. The gene expression level was calculated by Cufflinks (version 2.2.1) 50. The relative abundance of transcripts was measured as FPKM. Analysis of the differential gene expression between different groups was performed using the Cuffdiff module, and genes with a q-value of <0.05 were selected as being differentially expressed.

**2.7 Lysine crotonylation global analysis of PCOS-like mice ovarian tissues using mass spectrometry**

Ovarian tissue samples, from both mouse groups, were prepared by protein extraction, trypsin digestion, and affinity enrichment. For LC-MS/MS analysis, the resulting peptides were desalted using C18 ZipTips (Millipore, ZTC18S960) according to the manufacturer’s instructions.

After dissolution in solvent A (0.1% formic acid, 2% acetonitrile/in water), the tryptic peptides were loaded onto a homemade reversed-phase analytical column (25-cm length, 75/100 μm i.d.). Peptides were separated with a gradient from 6% to 24% solvent B (0.1% formic acid in acetonitrile) over 70 min, 24% to 35% over 14 min, and increasing to 80% over 3 min, then held at 80% for the last 3 min. A constant flow rate of 450 nL/min was maintained on a nanoElute UHPLC system (Bruker Daltonics, nanoElute® 2). The peptides were subjected to a capillary source, followed by timsTOF Pro mass spectrometry (Bruker Daltonics). The applied electrospray voltage was 1.60 kV. Precursors and fragments were analyzed using the TOF detector with an MS/MS scan range of 100 to 1700 m/z. The timsTOF Pro was operated in the parallel accumulation serial fragmentation (PASEF) mode. Precursors with charge states of 0–5 were selected for fragmentation, and 10 PASEF-MS/MS scans were acquired per cycle. The dynamic exclusion was set at 30 s.

The resulting MS/MS data was processed using the MaxQuant search engine (v.1.6.15.0). Tandem mass spectra were searched against the human SwissProt database (20422 entries) concatenated with the reverse decoy database. Trypsin/P was specified as the cleavage enzyme, allowing up to two missing cleavages. The mass tolerance for precursor ions was set to 20 ppm in the first search and 5 ppm in the main search, and the mass tolerance for fragment ions was set to 0.02 Da. Carbamidomethyl on Cys was specified as a fixed modification, and acetylation on the protein N-terminal and oxidation on Met were specified as variable modifications. FDR was adjusted to < 1%^57^.

**2.8 LONP1 knockdown with small interfering RNA (siRNA) and short hairpin RNA (shRNA) and plasmids preparation**

Endogenous LONP1 was knockdown using correspondingly targeted siRNA (c-149012) and control siRNA (sc-37007) were both purchased from Santa Cruz, USA. Stable knockdown of endogenous LONP1 was achieved using lentiviral vector harboring shRNA, which was first transfected into CHO cells, and then selected by puromycin of the infected cells.

The wild-type (WT) Lon protease homolog (LONP1) over-expressing plasmid and empty vector (EV) plasmid were constructed by amplifying the corresponding sequences and ligating them into pcDNA3.1-3xFlag-C vectors, respectively. Decrotonylated LONP1 mutants (K518R, K390R, K350R, and K363R) were generated by site-directed mutagenesis based on Lonp1-Wild type NP_083058.2. The WT and four site-mutant plasmids were contained synonymous mutation sequences related to siRNA and shRNA to avoid the knockdown effect of siRNA and shRNA targeting LONP1. All the sequences were verified by PCR amplification.

**2.9 Cell culture and transfections**

Chinese hamster ovary (CHO) cells and human embryonic kidney (HEK293T) cells were purchased from the National Biomedical Experimental Cell Resource Institute. Cells were cultured in DMEM containing 4.5g/L D-Glucose, 110 mg/L Sodium Pyruvate, L-Glutamine ( C11995500BT, Gibco), and 10% FBS (10099141, Gibco), at 37℃ in a 5% CO_2_ incubator. Cells were seeded at a density of 2 × 10^5^ viable cells mL^−1^, and passaged every 3–4 days.

For transient transfections, cells were grown to 70–90% confluence and transfected using Lipofectamine-3000 (L3000015, ThermoFisher Scientific) transfection reagents according to the manufacturer's instructions. Briefly, the lipofectamine mix, siRNA mix and plasmid mix were prepared separately, mixed, and incubated for 10 min at 20-25℃ before addition to the cells in Opti-MEM® medium.The transfected cells were incubated at 37°C and 5% CO2 for 48 h before harvested.

**2.10 Mitochondrial protein extraction**

Mitochondrial proteins were extracted from the transfected cells using a Minute mitochondrial isolation kit (MP-007, Invitrogen). The cells were pelleted by centrifugation at 1000rpm for 5 min and the supernatant discarded. The cell pellet was resuspended in 250 ul Buffer A by vortex and incubated on ice for 5-10 min. The supernatant was collected as a cytoplasmic fraction. Nuclei, and other components were removed by centrifugation at 16,000 × *g* for 30 seconds and resuspended in 200 µL of buffer B. Finally, after 30 minutes centrifugating at 16,000 × *g* the precipitate containing mitochondria was resuspended in mitochondrial protein lysate and used for in subsequent assays (co-immunoprecipitation (co-IP, western blot, and other experiments).

**2.11 Co-immunoprecipitation and western blotting**

Co-IP was performed on the extracted mitochondrial proteins (26147, Thermo Scientific). The extracted protein was purified using Pierce protein A/G agarose^58^. Pierce protein A/G agarose resin was bound to the spin column. Then 10 µg of flag antibody (anti-Flag (DYKDDDDK)-Tag, 66008-4-Ig, Proteintech) was added to the same column and washed. The cross-linked Flag antibody-bound spin column was retained. Pre-treated mitochondrial protein lysates with control agarose resin were then added to these columns and incubated overnight at 4℃. Finally, the columns were washed three times and the flow-through was stored for subsequent western blot experiments.

The Input and IP proteins were dissolved in the SDS-PAGE sample buffer. Equal amounts of the protein (10 µg) were separated by 10% SDS-polyacrylamide gel electrophoresis and transferred to PVDF membranes. The membrane was then washed with Tris-buffered saline, blocked for 1 h at 20-25℃, and incubated with the appropriate primary antibodies: anti-GAPDH (1:5000; 60004-1-Ig, Proteintech), anti-FLAG (66008-4-Ig, Proteintech), anti-LONP1 polyclonal (1:2000; 15440-1-AP, Proteintech) and anti-crotonyllysine mouse mAb (1:1000; PTM-502, PTM Bio). After washing, the cells were incubated with secondary antibody (horseradish peroxidase-conjugated goat anti-rabbit (1:5000, ZB2301, Zsbio), horseradish peroxidase-conjugated goat anti-mouse (1:5000, ZB2305, Zsbio)) for 1 h at 20-25℃. The blots were then visualized using SuperSignal-enhanced chemiluminescent substrate solution (WBULS0100, Millipore).

**2.12 RNA extraction and real-time quantitative PCR (RT‒PCR) analysis**

The mRNA level of LONP1 expression level was evaluated by RT-qPCR. The extraction of total RNA of cells was conducted after siRNA transfected for 48h. TRIzol (15596018, Gibco) was used for total RNA extraction according to manufacturer’s instructions. TransScript First-Strand cDNA Synthesis SuperMix (AT301-02, TransGen Biotech, China) was used for reverse transcription. RT-qPCR was performed using Fast SYBR Mixture (Low ROX) (CW2621M, CWBIO, China)

**2.13 Flow cytometry assay**

After the transfected cells were prepared into suspension, they were incubated with dyes: JC-1 (C2006, Beyotime, China) to determine mitochondrial health, and MitoSOX™ Red reagent (M36008, Invitrogen), to determine ROS activity, for 30 min at 37°C. The cells were washed three times with 1× phosphate-buffered saline (PBS) buffer (pH 7.4). The cells were monitored, by flow cytometry on using a FACSAria II flow cytometer (BD Biosciences, San Jose, CA, USA). The resulting data was analyzed using FlowJo software.

**2.14 Immunocytochemistry**

GV and MII oocytes were fixed in 4% paraformaldehyde for 10 minutes at 20-25℃ and then transfer the oocytes to 0.1% Ttiton-X100 solution for 20 min. After that, 5% BSA was needed for an hour. After incubation with anti-LONP1 (1:500; ab22431, abcam, United Kingdom) overnight at 4 °C. Then, the oocytes were washed in PBS and incubated with secondary antibodies. The nuclei were counterstained with DAPI

(C1002, Beyotime, China). Results were observed under a laser scanning confocal microscope.

**2.15 Blood collection from PCOS woman for clinical evaluation**

The study subjects consisted of PCOS patients and healthy women who visited the Peking Union Medical College Hospital, Department of Obstetrics and Gynecology, Endocrinology, and Reproductive Center between October 2021 and February 2022. PCOS was diagnosed according to the 2003 Rotterdam Criteria, that is, the presence of two or more oligo-ovulation and/or anovulation cycles, clinical and/or biochemical signs of hyperandrogenism, and polycystic ovaries after exclusion of other etiologies. The control subjects were healthy women randomly recruited from society. PCOS patients we enrolled in were not previously diagnosed and received treatments related to PCOS. Eighteen pairs of healthy females and patients with PCOS were included in the study, and peripheral blood samples were collected from each participant. The following indicators were measured: height, body weight, body mass index (BMI), ultrasound of the uterus and bilateral ovaries, serum levels of testosterone, LH, FSH, triglycerides, total cholesterol, high-density lipoprotein cholesterol, low-density lipoprotein cholesterol, fasting serum glucose, and insulin. Participants who had breastfed, become pregnant within the past year, or taken medication within the past 3 months were excluded from the study. The patient characteristics are listed in **Table 1**. This study was approved by the Research Ethics Board of Peking Union Medical College Hospital (JS-3213) and was performed in accordance with the World Medical Association Declaration of Helsinki. All subjects included in the study signed a declaration of informed consent.

**2.16 Whole blood protein extraction**

The human whole blood protein extraction kit (31406, Beibo, China) was used to extract protein from the blood samples according to the manufacturer’s instructions. Protease inhibitor mixture (1X) was added to every protein extraction solution, mixed, and put on ice for later use. Whole blood (300 µL) was mixed with 300 µL protein extract and vortexed at 4°C for 40 min. Then, centrifuged at 14,000 × *g* for 10 min in a pre-cooled centrifuge (4°C), and the supernatant contained the whole blood protein.
